# Influenza-like illness symptoms due to endemic human coronavirus reinfections are not influenced by the length of the interval separating reinfections

**DOI:** 10.1128/spectrum.03912-23

**Published:** 2024-02-08

**Authors:** Ferdyansyah Sechan, Arthur W. D. Edridge, Jacqueline van Rijswijk, Maarten F. Jebbink, Martin Deijs, Margreet Bakker, Amy Matser, Maria Prins, Lia van der Hoek

**Affiliations:** 1 Laboratory of Experimental Virology, Department of Medical Microbiology and Infection Prevention, Amsterdam UMC, University of Amsterdam, Amsterdam, the Netherlands; 2 Amsterdam Institute for Infection and Immunity, Amsterdam, the Netherlands; 3 Amsterdam Public Health, Amsterdam, the Netherlands; 4 Netherlands Institute for Health Services Research (NIVEL), Utrecht, the Netherlands; 5 Department of Infectious Diseases, Amsterdam UMC, University of Amsterdam, Amsterdam, the Netherlands; 6 Department of Infectious Diseases, Public Health Service of Amsterdam, Amsterdam, the Netherlands; National Chung Hsing University, Taichung, Taiwan

**Keywords:** SARS-CoV-2, HCoV-NL63, HCoV-29E, HCoV-OC43, HCoV-HKU1, reinfections, influenza-like illness

## Abstract

After 3 years of its introduction to humans, severe acute respiratory syndrome coronavirus 2 (SARS-CoV-2) has been declared as endemic. Little is known about the severity of the disease manifestation that future infections may cause, especially when reinfections occur after humoral immunity from a previous infection or vaccination has waned. Such knowledge could inform policymakers regarding the frequency of vaccination. Reinfections by endemic human coronaviruses (HCoVs) can serve as a model system for SARS-CoV-2 endemicity. We monitored 44 immunocompetent male adults with blood sampling every 6 months (for 17 years), for the frequency of HCoV (re-)infections, using rises in N-antibodies of HCoV-NL63, HCoV-29E, HCoV-OC43, and HCoV-HKU1 as markers of infection. Disease associations during (re-)infections were examined by comparison of self-reporting records of influenza-like illness (ILI) symptoms, every 6 months, by all participants. During 8,549 follow-up months, we found 364 infections by any HCoV with a median of eight infections per person. Symptoms more frequently reported during HCoV infection were cough, sore throat, and myalgia. Two hundred fifty-one of the 364 infections were species-specific HCoV-reinfections, with a median interval of 3.58 (interquartile range 1.92–5.67) years. The length of the interval between reinfections—being either short or long—had no influence on the frequency of reporting ILI symptoms. All HCoV-NL63, HCoV-229E, HCoV-OC43, and HCoV-HKU1 (re-)infections are associated with the reporting of ILIs. Importantly, in immunocompetent males, these symptoms are not influenced by the length of the interval between reinfections.

**IMPORTANCE:**

Little is known about the disease following human coronavirus (HCoV) reinfection occurring years after the previous infection, once humoral immunity has waned. We monitored endemic HCoV reinfection in immunocompetent male adults for up to 17 years. We found no influence of reinfection interval length in the disease manifestation, suggesting that immunocompetent male adults are adequately protected against future HCoV infections.

## INTRODUCTION

Severe acute respiratory syndrome coronavirus 2 (SARS-CoV-2), the causative agent of the coronavirus disease of 2019 (COVID-19), is a *Betacoronavirus* that entered the human population in 2019 (1). Following an infection, protective immunity wanes within a year and reinfections occur, which is also influenced by newly rising variants (2). At the same time, subsequent infections with SARS-CoV-2 appeared to result in a less severe COVID-19 disease regardless of the variant of the primary infection (3). Frequent reinfections and reduced severity of the disease are signs that this virus cannot be eradicated and is now endemic.

Understanding whether the level of disease protection remains after a certain amount of time has elapsed since the previous viral infection is relevant to infectious disease control, in particular for science-based policies on vaccinations. A yearly vaccination strategy against a virus has been considered to replenish immunity. Such a vaccination strategy is costly, and long-term data on the impact of vaccination on viral spread, optimal timing, and the dynamics with natural reinfections are not there yet, as SARS-CoV-2 entered the population just 3 years ago. Studies on SARS-CoV-2 reinfections are limited to events occurring after 3 months, based on a reinfection definition, until a maximum of 3 years (4). It remains unknown whether subsequent SARS-CoV-2 infection that occurs more than 3 years after the first infection results in a different disease manifestation than shorter ones. Related viruses, such as the endemic human coronaviruses (HCoVs), can be studied to gain insight into disease manifestation during reinfections that occur multiple years (more than three) after a previous infection.

Four HCoV species have been circulating in humans for centuries: HCoV-NL63, HCoV-229E, HCoV-OC43, and HCoV-HKU1. Animal-infecting coronaviruses with close relatives with these HCoVs have been found, suggesting zoonotic origins (5). HCoV-NL63 and HCoV-229E belong to the *Alphacoronavirus*, while HCoV-OC43 and HCoV-HKU1 belong to the genus *Betacoronavirus,* such as SARS-CoV-2. HCoV-NL63 and SARS-CoV-2—although belonging to different genera—share one important characteristic, being that they both use angiotensin-converting enzyme 2 to enter their target cells (1, 6). The particular symptoms caused by each HCoV are not fully known. Experimental infections by HCoV-229E and HCoV-OC43 on healthy volunteers between 1960 and 1990 revealed common respiratory symptoms, such as cough, sore throat, myalgia, and runny nose (7).

We have previously shown that reinfection by any of the HCoVs occurs fast, sometimes as early as within 6–12 months, with a median time to reinfections being around 36 months (8). Repeat experimental infection studies with HCoV-229E on healthy individuals revealed mild or no symptoms upon reinfections after a year (9, 10). No experimental infection, or natural infection study, has investigated the clinical presentation of an HCoV reinfection occurring more than 1 year after a previous infection. A study on the relationship of the interval length between reinfections and disease characteristics of HCoVs can provide important knowledge on the potential longitudinal patterns of disease manifestation of SARS-CoV-2 as long as follow-up data for this new virus are not there yet.

In the current study, we investigated the relationship between reporting of influenza-like illness (ILI) symptoms and infection with any or each specific HCoV. Subsequently, we investigated the relationship between the reporting of ILI symptoms and the length of the interval between reinfections by the same HCoV species. The study was conducted among immunocompetent male volunteers who were followed for a median of 16 years (with the follow-up ranging between 7 and 17 years).

## MATERIALS AND METHODS

### Study design and population

Amsterdam Cohort Studies (ACS) investigates the epidemiology of HIV-1 and other sexually and blood-transmitted infections and the development of their associated illnesses among men who have sex with men, either with or without HIV-1 infection. The inclusion criteria of this study include being over 18 years of age, taking part in sexual activities with other men 6 months prior to recruitment, and residing in Amsterdam (11). Subjects participated voluntarily, provided written informed consent, and received no monetary incentive. HIV-1-negative subjects were followed up every 6 months, from whom blood samples were collected, and serum samples from these visits were stored at −80°C until further use. During study visits, a standardized questionnaire documenting sociodemographics and sexual behavior was completed. Subjects were also asked by the study staff about the presence of ILI symptom(s) in the preceding 6 months or since the last study visits for the purpose of assessing the possibility of acute HIV-1 infection. The recorded ILI symptoms included fever (>38°C for more than 3 days), cough, dyspnea, fatigue, headache, sore throat, myalgia, nausea, and diarrhea (watery stool for a minimum of 7 days). Other information pertaining demographics relevant to ILI research or other measurements on awareness or prevention of respiratory illness was not asked by the study nurse and thus not documented. The study was approved by the Medical Ethics Committee of the Amsterdam University Medical Center of the University of Amsterdam, the Netherlands (MEC 07/182).

Forty-four adult males were included in this study from ACS on HIV-1 infection among men who have sex with men (11). The inclusion criteria for these subjects include a minimum of 7 years of observation from January 2003 onward and remaining HIV-1 negative throughout the follow-up.

### Partial HCoV nucleocapsid enzyme-linked immunosorbent assay

The enzyme-linked immunosorbent assay (ELISA) using NL63-NCt, 229E-NCt, OC43-NCt, and HKU1-NLCt antigens was conducted as previously described (8, 12). The C-terminus of the viral nucleocapsid protein (NCt) was used, except for HCoV-HKU1, for which the antigen consisted of both the linker (L) and the Ct domain of the viral protein (12). The cross-reactivity evaluation of HKU1-NLCt antigen against HCoV-OC43 is described in Table S1 and S2. All serum samples from one subject were grouped within one ELISA plate and tested in duplicate (duplicates on different plates). The antibody level in a given time point is expressed as the geometric mean of duplicates in relative luminescence unit.

### Data analysis

Antibody dynamic was reported as ELISA signal fold-change between two subsequent time points. HCoV infection is defined as the fold-change values of 1.40 or higher for each HCoV antigen, and the time points in which such rise was observed were designated as the infection date (8). A cut-off of a minimum 10% difference between fold-change values in one time point was implemented to account for possible cross-reactivity within the HCoV genus (HCoV-NL63 and HCoV-229E, or HCoV-OC43 and HCoV-HKU1) (8). The association between having an infection by any or each HCoV and reporting of symptom(s) within the last 6 months was first explored with cross-tabulation and later modeled using univariable logistic regression analysis with generalized estimating equations (GEEs) with an exchangeable correlation matrix. We opted for the GEE approach due to the repeated-measure structure of the data (each individual was observed multiple times), and the binary outcome is selected to account for predicting the outcome of the model (symptom presence yes or no). Data points with infection with an HCoV species other than the HCoV species under modeling were excluded from the data set. For example, in the models for HCoV-NL63-infection, the data points positive for this virus were included, but the negative data points (no HCoV-NL63 infection) were only included if no infection by one or more of the other three HCoVs was found. Data points with no infection were thus assigned as the reference category. All models were corrected for age at the sampling date in a multivariable analysis.

Reinfections were defined as an infection following another infection by the same HCoV species. Interval length between reinfections was defined as the interval between sampling dates on which an infection was established. The reinfection interval length between HCoVs was compared by the Kruskal-Wallis test (a Dunn’s multiple-comparison *post hoc* test). The association between the length of an HCoV reinfection interval in years and the reporting of symptom(s) within the last 6 months before the end of the said interval was first explored with cross-tabulation and later modeled using univariable logistic regression analysis with GEE. All reinfection intervals were pooled in one data set to generate the statistical model. This interval length model was also used in a multivariable analysis looking at the presence of infections by other HCoVs that belong to the same genus (HCoV-NL63 and HCoV-229E in the genus *Alphacoronavirus* or HCoV-OC43 and HCoV-HKU1 in the genus *Betacoronavirus*).

Statistical analyses were conducted using Stata version 18.0 (StataCorp). The association is presented as odds ratio (OR) and 95% confidence interval (CI). The *P*-value of each independent variable in GEE was calculated, and *P* < 0.05 was considered significant.

## RESULTS

We analyzed 1,378 serum samples from 44 healthy adult males from the ACS in 8,549 follow-up months (712.4 years). Median follow-up was 199 months [interquartile range (IQR), 196–208 months]. At baseline, their median age was 31 years (IQR 28–36 years). We found 364 infections by any HCoV (Fig. S1; Table S3), with a median of 8 infections per person (IQR 6–10 infections per person). HCoV-OC43 infections were observed the most (*n* = 143), followed by HCoV-229E (*n* = 132), HCoV-NL63 (*n* = 79), and HCoV-HKU1 (*n* = 52). For 1,335 visits, HCoV infection status and ILI symptoms were known. These included 971 visits without any HCoV infection and 364 visits with infection. The symptoms more frequently reported at visits with infection by any HCoV than at visits with no HCoV infection were cough, sore throat, and myalgia. Cough was reported at 85 (6%) visits, and in 36 (42%) of these visits, infection by an HCoV was found. Sore throat was reported at 133 (10%) visits, and 44 (33%) of these visits were with an HCoV infection. Myalgia was reported at 71 (5%) visits, and 31 (44%) of these visits were with an HCoV infection (Table 1).

**TABLE 1:** Frequency and percentage of each symptom being reported for any or each infecting HCoV

Symptom	No HCoV		Any HCoV		HCoV-NL63		HCoV-229E		HCoV-OC43		HCoV-HKU1	
	*n*	(*%*)^*a*^	*n*	(%)^*a*^	*n*	(%)^*a*^	*n*	(%)^*a*^	*n*	(%)^*a*^	*n*	(%)^*a*^
*N*	971		364		79		132		143		52	
Fever	74	(7.6)	38	(10.4)	14	(17.7)	11	(8.3)	13	(9.1)	7	(13.5)
Cough	49	(5.1)	36	(9.9)	11	(13.9)	12	(9.1)	14	(9.8)	5	(9.6)
Dyspnea	24	(2.5)	9	(2.5)	3	(3.8)	3	(2.3)	2	(1.4)	1	(1.9)
Fatigue	102	(10.5)	46	(12.4)	11	(13.9)	13	(9.9)	21	(14.7)	5	(9.6)
Headache	50	(5.2)	21	(5.8)	7	(8.9)	6	(4.6)	8	(5.6)	3	(5.8)
Sore throat	89	(9.2)	44	(12.1)	11	(13.9)	14	(10.6)	18	(12.6)	4	(7.7)
Myalgia	40	(4.1)	31	(8.5)	6	(7.6)	11	(8.3)	13	(9.1)	6	(11.5)
Nausea	45	(4.6)	17	(4.7)	6	(7.6)	7	(5.3)	2	(1.4)	3	(5.8)
Diarrhea	59	(6.1)	17	(4.7)	5	(6.3)	7	(5.3)	11	(7.7)	4	(7.7)

^*a*^ Percentage was calculated based on the presence of the symptom within all data points in each group.

**Fig 1:**
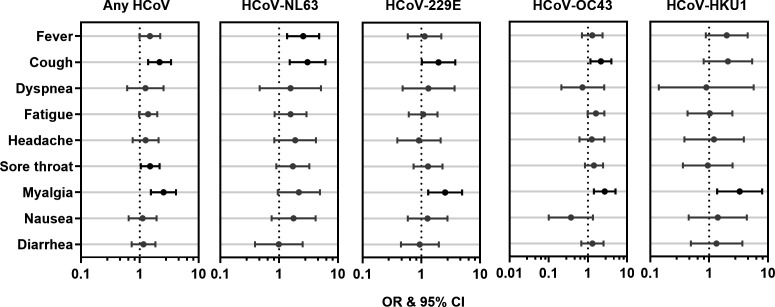
HCoV infection is associated with the reporting of some ILI symptoms. Forest plots describing the OR and 95% CI for any HCoV (leftmost panel) and each HCoV (second-to-fifth panel from the left). OR and 95% CI values were generated from univariable analyses and are presented on a logarithmic scale for each ILI symptom. The black point and bar indicate a significant association. The dotted lines represent no association (OR = 1).

Univariable analysis revealed significant associations between the aforementioned symptoms and HCoV infection: cough (OR 2.18; 95% CI 1.39–3.41), sore throat (OR 1.5; 95% CI 1.04–2.17), and myalgia (OR 2.54; 95% CI 1.55–4.15) (Fig. 1, leftmost panel). Some symptoms were associated with individual HCoVs (Fig. 1, second-to-fifth panels from the left). Fever was associated with HCoV-NL63 (OR 2.57; 95% CI 1.37–4.82). Cough was associated with HCoV-NL63 (OR 3.05; 95% CI 1.52–6.14), HCoV-229E (OR 1.96; 95% CI 1.02–3.72), and HCoV-OC43 (OR 2.15; 95% CI 1.16–3.98). Myalgia was associated with HCoV-229E (OR 2.55; 95% CI 1.31–4.94), HCoV-OC43 (OR 2.7; 95% CI 1.43–5.10), and HCoV-HKU1 (OR 3.32; 95% CI 1.37–8.00). After adjusting for age, most aforementioned significant associations remained except for fever and HCoV-NL63 (adjusted OR 1.80; adjusted 95% CI 0.76–4.29) (Table S4). Dyspnea, fatigue, headache, nausea, and diarrhea were not significantly associated with infection by any or each HCoV.

**Fig 2:**
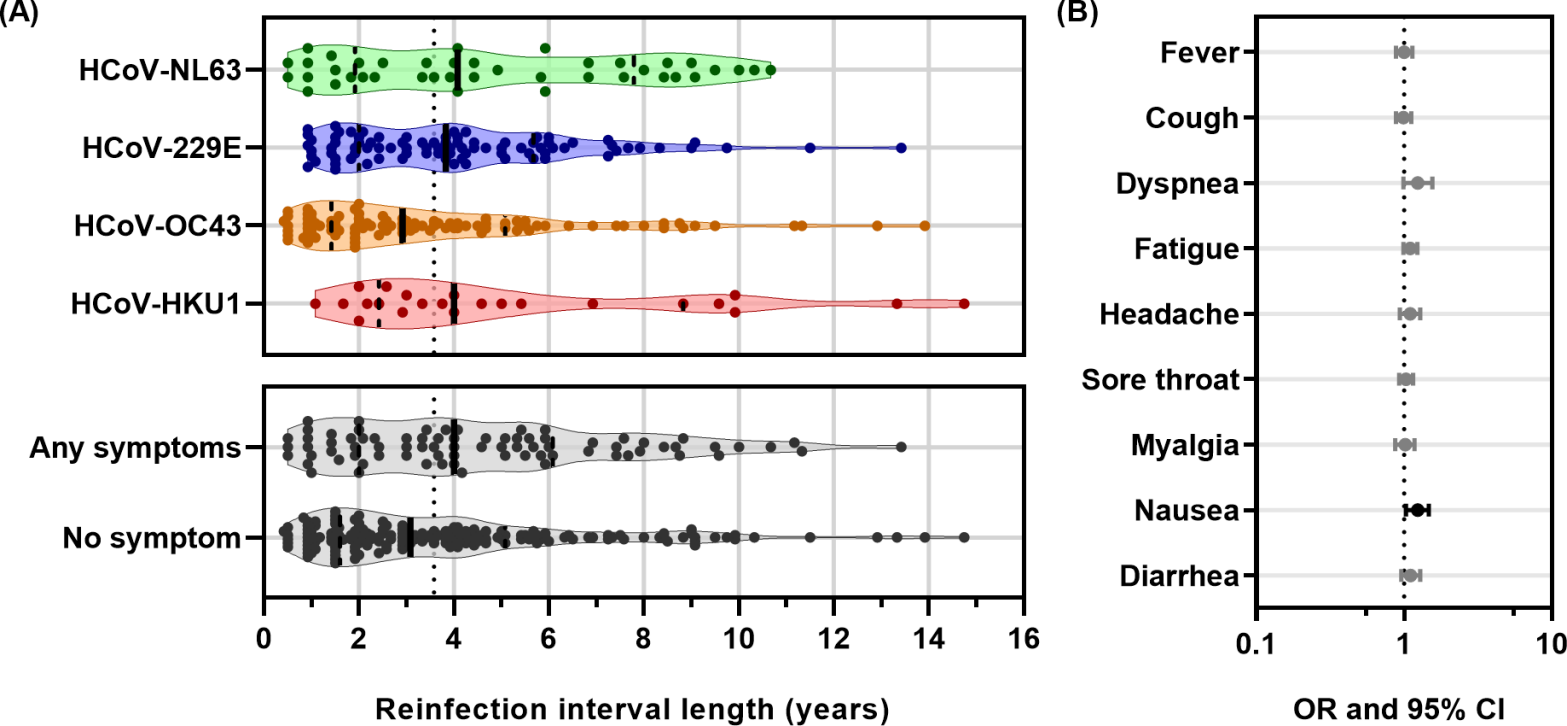
Reinfection interval length did not affect the reporting of ILI-like symptoms. (**A**) Violin plot expressing the distribution of interval length in years and categorized based on the infection by each endemic HCoV (upper panel) or the absence/presence of any ILI symptom (lower panel). Each dot represents one interval. The solid vertical line in each data set: median length of each data set. Dashed vertical line: 25% and 75% percentile of each data set. Dotted vertical line: median length of all intervals (3.58 years). (**B**) Forest plots describing the OR and 95% CI for any HCoV. OR and 95% CI values were generated from univariable analyses and are presented on a logarithmic scale for each ILI symptom. The black point and bar indicate significant association. The dotted lines represent no association (OR = 1).

Reinfections by the same HCoV species were found (Table S3). In total, 251 out of 364 HCoV infections (69%) were observed as reinfections. There were 42 HCoV-NL63, 86 HCoV-229E, 100 HCoV-OC43, and 23 HCoV-HKU1 reinfections. The median interval length until observed reinfection was 4.08 (IQR 2.00–7.58) years for HCoV-NL63, 3.83 (IQR 2.00–5.67) years for HCoV-229E, 2.92 (IQR 1.46–4.88) years for HCoV-OC43, and 4.00 (IQR 2.04–6.04) years for HCoV-HKU1 (Fig. 2A, upper panel). The distribution of interval length differed between the four endemic HCoVs (*P* = 0.0457), and yet, the *post hoc* analysis did not detect a statistically significant difference between any combination of two endemic HCoV interval lengths (*P* values between 0.109 and 0.999). When all reinfection intervals were grouped together, the median interval length until observed reinfection was 3.58 (IQR 1.92–5.67) years (Fig. 2A, vertical dashed line).

There were 77 (31%) reinfection intervals at which at least one symptom was reported. The interval length did not differ significantly between reinfections with and without symptoms (*P* = 0.052) (Fig. 2A, bottom panel). Using univariable analysis between interval length in years and symptom reporting, we only found a significant association for nausea (OR 1.24; 95% CI 1.04–1.47) (Fig. 2B). However, nausea was reported very rarely, only in 10 (3.9%) of all reinfections (Table S5). None of the other symptoms was significantly associated with the increasing interval length.

We examined whether there was evidence for cross-protection for HCoV infections belonging to the same genus (the *Alphacoronaviruses* HCoV-229E and HCoV-NL63 or the *Betacoronaviruses* HCoV-OC43 and HCoV-HKU1). Using the chi-square test, we found that intragenus HCoV infections were observed in less than half (*n* = 81) of all reinfection intervals as opposed to a hypothetical scenario when the chance of intragenus infection found was 50:50 (*P* < 0.001). We also noted that the reinfection intervals with observed infection by an intragenus HCoV were longer compared to intervals without within-genus-HCoV infection (*P* < 0.001) (Fig. S2). Subsequently, we investigated whether the association between interval length and symptoms differed for intervals with or without within-genus HCoV infection. No statistical evidence of such difference in association was found for each symptom.

## DISCUSSION

In this study, we found associations between the reporting of ILI-like symptoms such as cough and myalgia and an infection by any endemic HCoV in healthy adult males in the Netherlands. Cough and myalgia were significantly associated with an infection by three out of four HCoV species. The waning humoral immunity that has been observed in HCoV infections (8, 10) raised a question: would an extended time (several years) between infections result in a different disease manifestation? Our analysis showed that the reinfection interval length did not influence the occurrence of ILI symptoms, except nausea, which was, however, a rarely reported symptom in the first place. Therefore, our data suggest that there is no adverse outcome from subsequent HCoV infection that happens more than 3 years after the previous one. We hypothesize that instead of circulating antibodies, T- and B-cell memory may be the most important component acting in HCoV reinfections.

Comparison of disease manifestation between an HCoV infection and a subsequent one (same species) was originally done with experimental infection studies. In these studies, healthy adults were purposely infected with virus culture and observed afterward. Such studies have been conducted with HCoV-229E (10, 13), with the same subjects being experimentally reinfected 1 year after the first. These studies reported that subsequent reinfections resulted in either no disease or similar to the first one. Another approach to studying reinfections and disease is by observing a cohort longitudinally. Our study benefitted from the longitudinally collected data spanning up to 17 years for most subjects. There is only one other study, spanning 2 years, by Galanti and Shaman, who observed no change in severity in the subsequent infection by endemic HCoVs compared to the first one (14).

An interesting question to answer is whether a first infection by an HCoV would influence the disease of a subsequent infection by a different HCoV from the same genus (e.g., an HCoV-NL63 infection followed by HCoV-229E infection). Each endemic HCoV reaches peak infections every 2 years with alternative peaks between HCoVs from the same genus (15, 16), suggesting such interactions. Furthermore, in a population of healthcare workers, signs of recent HCoV-OC43 infections were associated with having lower odds of SARS-CoV-2 infection (17). Such intergenus protection could possibly be mediated by some cross-reactivity from shared epitopes (18–20). We analyzed intergenus infections in the reinfection intervals yet found no signs that ILI symptoms were less reported in those cases.

Our study had some limitations. The ILI symptoms reported at each follow-up were an accumulation of all symptoms experienced in the past 6 months. The reporting of these symptoms was also done retrospectively, and the self-reporting approach used in this study could be prone to recall bias. Details on the severity of the ILI-like symptoms were also not scored, except for the presence or absence of them. This is due to the cohort originally being designed to study HIV-1 transmission in communities, and ILI resembles symptoms of primary HIV-1 infection. In a study on respiratory infections, a diary system implemented when the subjects experience symptoms would be able to capture the disease manifestation more accurately. Such an experimental setup would also allow further analysis of the severity of symptoms. Next to that, an extensive record on relevant demographics (occupation, income range, household size, presence of children in the household, number of frequent contacts outside household, frequency of using public transport, etc.) and awareness or prevention of catching respiratory diseases, such as face masking, hand washing procedure after sneezing or coughing, nose-picking, and handkerchief usage was not logged. Future studies are advised to document such information in (long term) ILI-related studies. It also needs to be mentioned that our study population consisted exclusively of adult men. Sex appeared to influence the outcome of COVID-19 disease at the start of the pandemic (21). We, therefore, strongly encourage future studies to include women. Our study participants were all men who had sex with men who participated voluntarily in an HIV-1 risk study. They could be more aware of health issues and may remember and report better on past ILIs than the average person in the Netherlands. Lastly, we might have missed some HCoV-HKU1 infections in our population. Past research in our group revealed that HCoV-HKU1 infection resulted in a less robust humoral immunity response compared to the other three HCoVs (8, 12), and reinfections by this virus might have been missed.

In summary, we did not find that a long interval between infections had an effect on the presence of ILI symptoms our study participants experienced during that reinfection. Our data suggest that the immunocompetent adult male population is adequately protected during subsequent HCoV infection. Apparently, the absence of immune-boosting (either on HCoV species level or on HCoV genus level) does not make people more frequently ill when they eventually do catch the virus. It is unknown when SARS-CoV-2 will become fully adapted to humans like the HCoVs are now. However, once the virus has been fully adapted, and the vast majority of people having been infected at least once, our findings—in immunocompetent male adults—could suggest that there may not be an urgent need to repeatedly vaccinate the general immunocompetent population against the virus.

## SUPPLEMENTAL MATERIAL

Supplemental figures spectrum.03912-23-s0001.pdf Fig. S1 and S2.

Supplemental text and tables spectrum.03912-23-s0002.docx Supplemental materials and methods and Tables S1 to S5.

ASM does not own the copyrights to Supplemental Material that may be linked to, or accessed through, an article. The authors have granted ASM a non-exclusive, world-wide license to publish the Supplemental Material files. Please contact the corresponding author directly for reuse.
