# Supplementary material for: Influenza-like illness symptoms due to endemic human coronavirus reinfections are not influenced by the length of the interval separating reinfections
Source: Microbiol Spectr. 2024 Feb 8;12(3):e03912-23. doi: 10.1128/spectrum.03912-23 (PMC10913438; doi:10.1128/spectrum.03912-23)

Supplementary Material

Influenza like illness symptoms due to endemic human coronavirus reinfections are not influenced by the length of the interval separating reinfections

Ferdyansyah Sechan[1,2], Arthur W. D. Edridge[1,2], Jacqueline van Rijswijk[1,2], Maarten F. Jebbink[1,2], Martin Deijs[1,2], Margreet Bakker[1,2], Amy Matser[2,3,4], Maria Prins[2,5,6], Lia van der Hoek*[1,2]

1 Laboratory of Experimental Virology, Department of Medical Microbiology and Infection Prevention, Amsterdam UMC, University of Amsterdam, Amsterdam, Netherlands.
2 Amsterdam Institute for Infection and Immunity, Amsterdam, Netherlands.
3 Amsterdam Public Health, Amsterdam, Netherlands
4 Netherlands Institute for Health Services Research (NIVEL), Utrecht, the Netherlands
5 Department of Infectious Diseases, Amsterdam UMC, University of Amsterdam, Amsterdam, the Netherlands
6 Department of Infectious Diseases, Public Health Service of Amsterdam, Amsterdam, the Netherlands

*__Correspondence:__ Lia van der Hoek (c.m.vanderhoek@amsterdamumc.nl)

This file includes

- Supplementary Figure 1,2

Supplementary Figure 1. Antibody dynamics of partial nucleocapsid antigen of endemic HCoV throughout time for subject 1 - 44. *Data is presented as geometric mean ± SD of two technical replicates for each serum sample in Relative Luminescence Unit (RLU). White dots: fold-change below 1.4. Black dots: fold change ≥ 1.40 as the indication of infection with fold change values given in black and bold above the data points. Gray dots: fold change ≥ 1.40 but not counted as infection due to either being cross-reaction (within-genus fold-change difference > 10%) or no difference could be made (within-genus fold-change difference: ≤ 10%); fold change values are given in black and italic above the data points.*

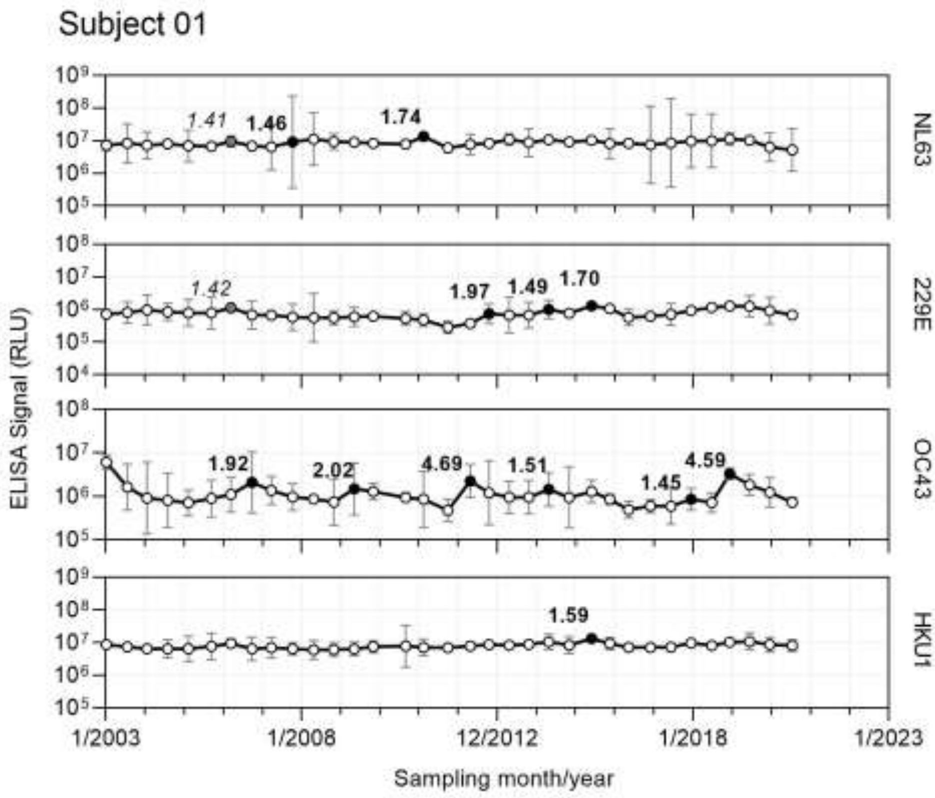

Supplementary Figure 1 (continued)

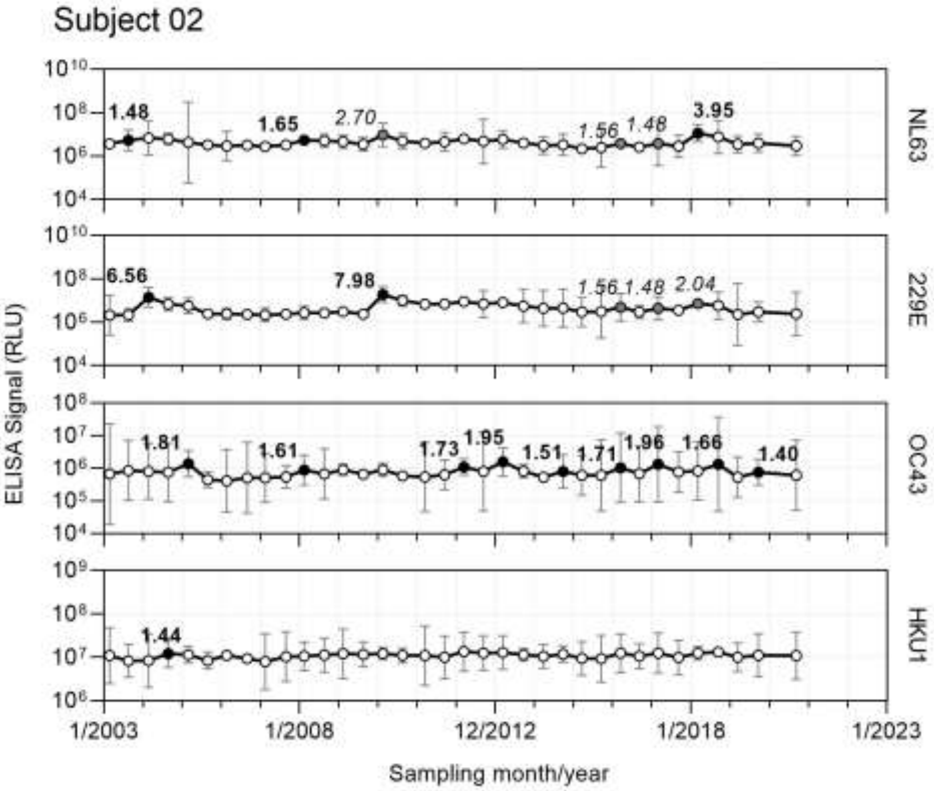

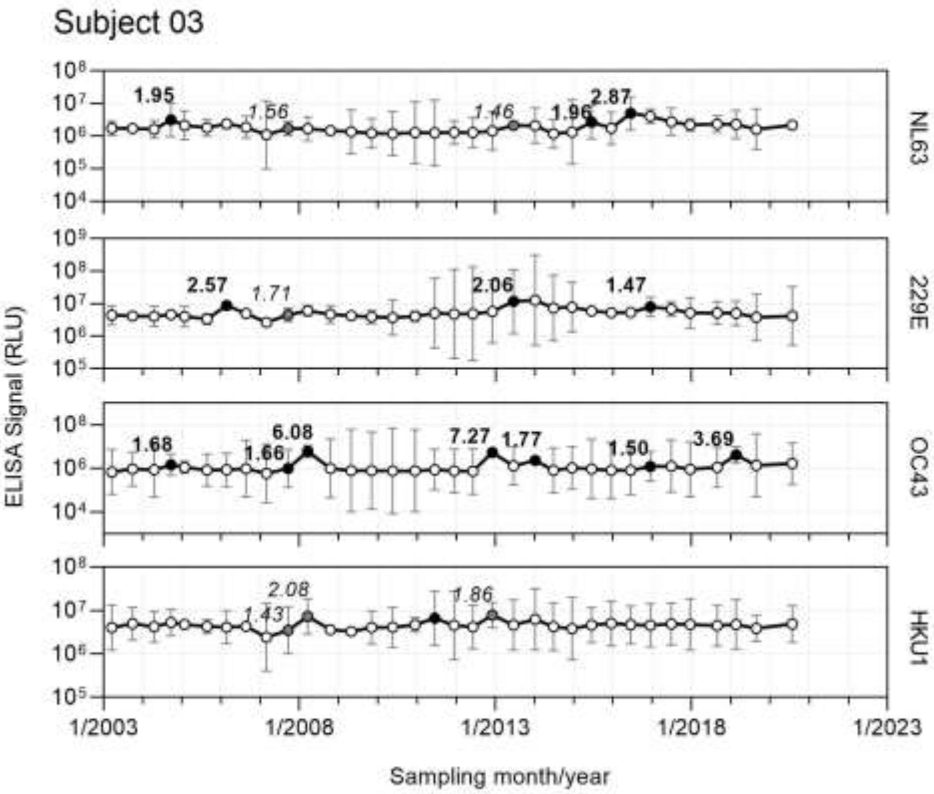

Supplementary Figure 1 (continued)

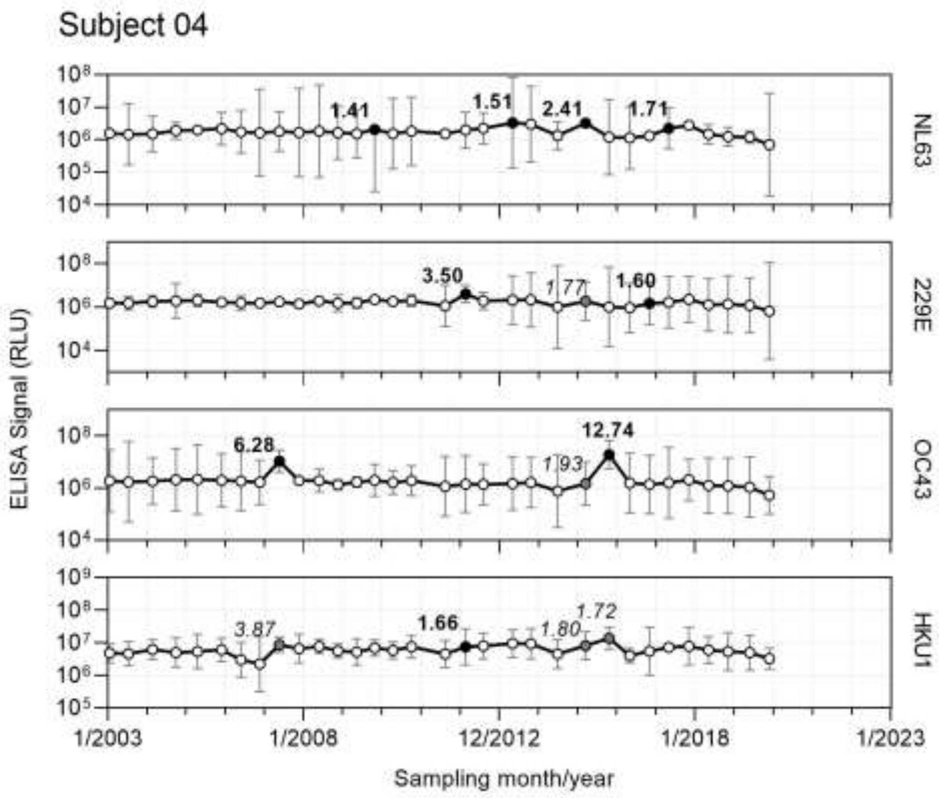

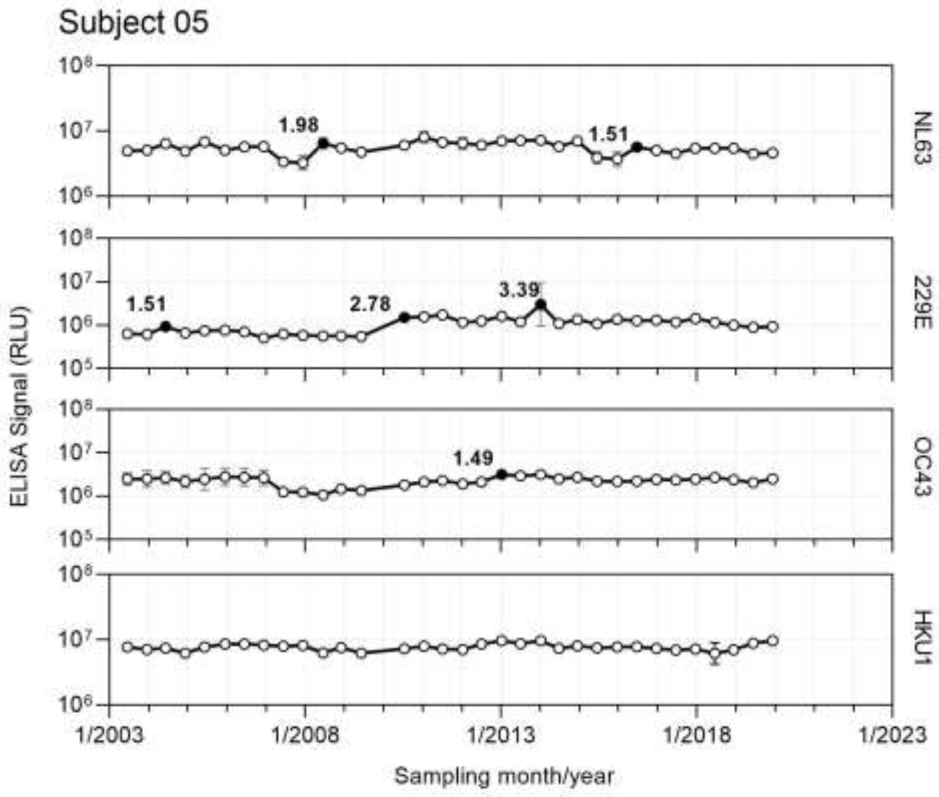

Supplementary Figure 1 (continued)

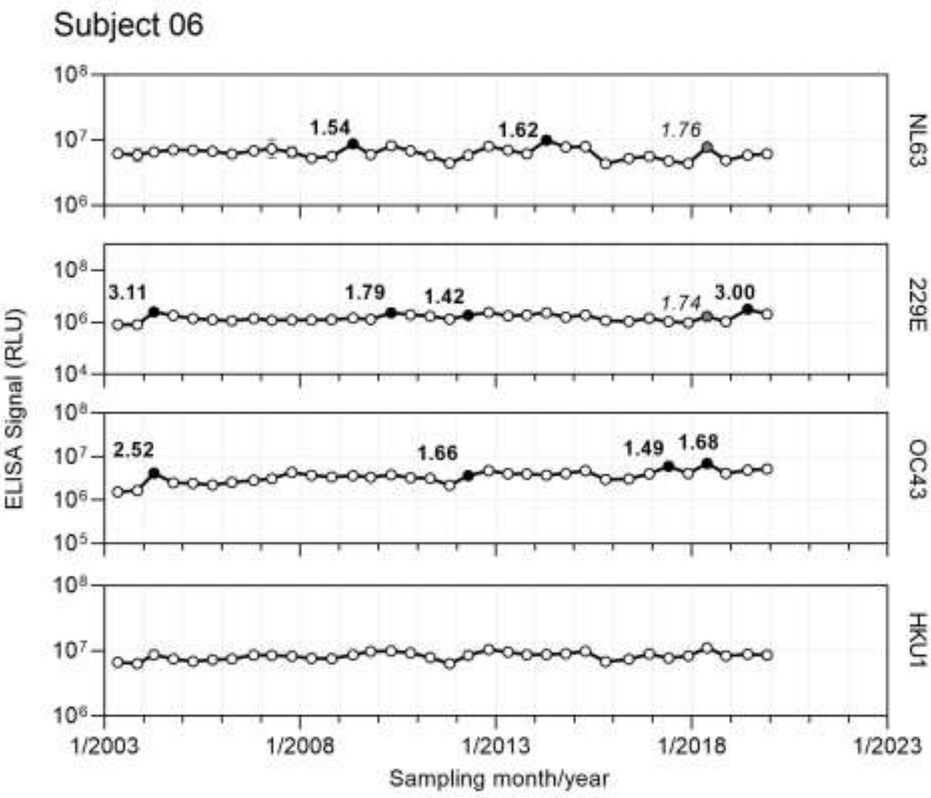

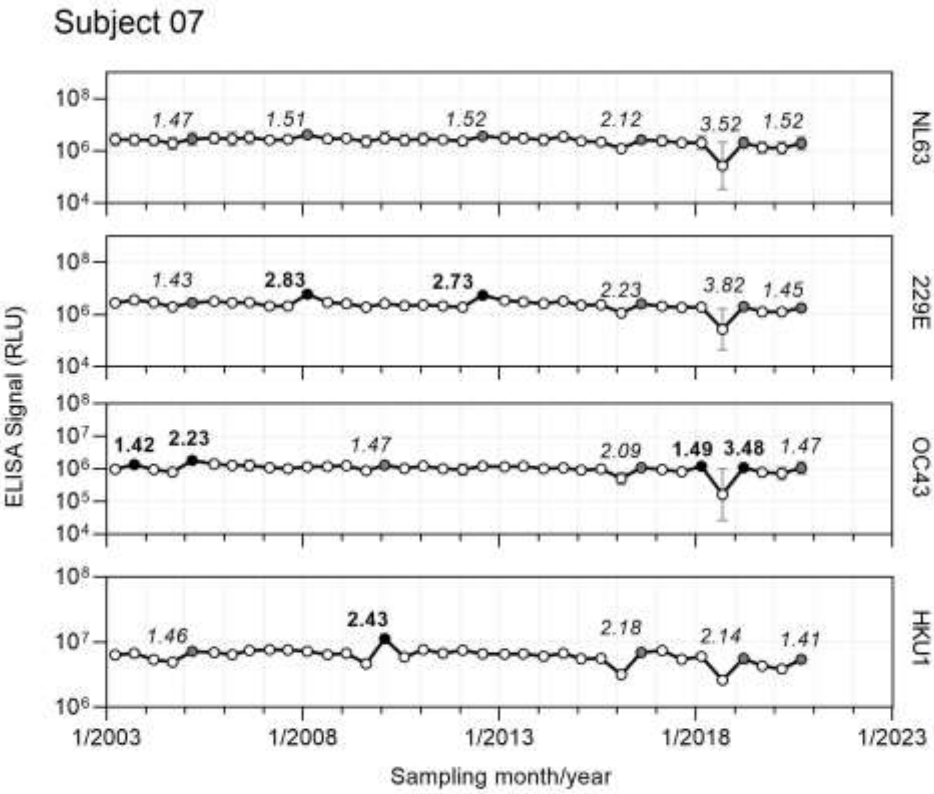

Supplementary Figure 1 (continued)

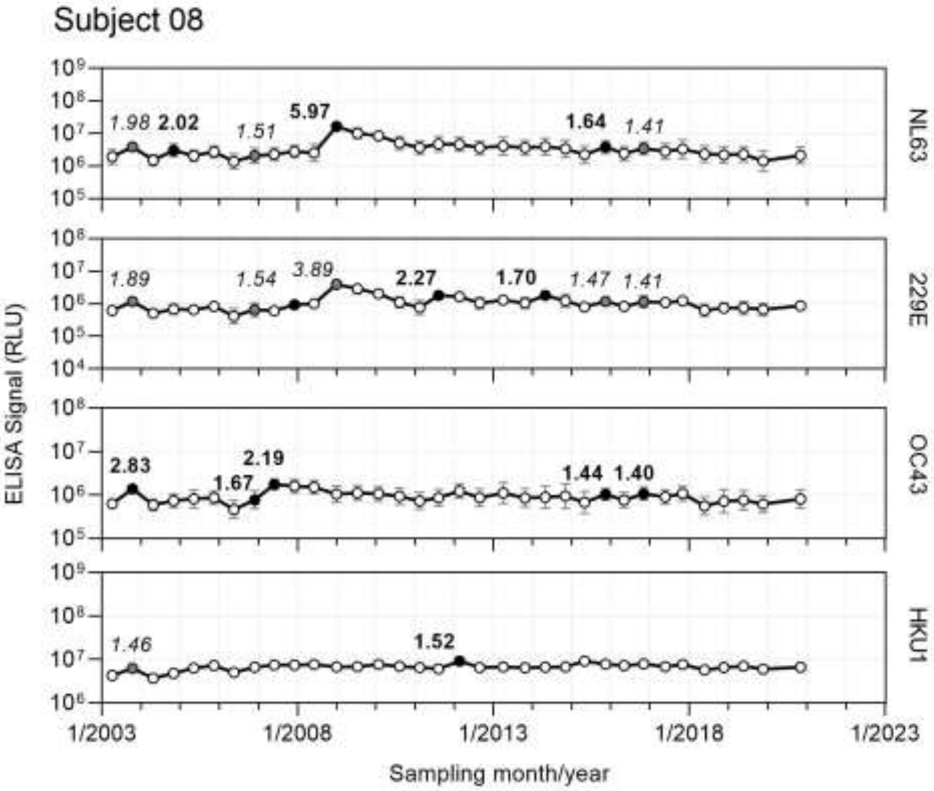

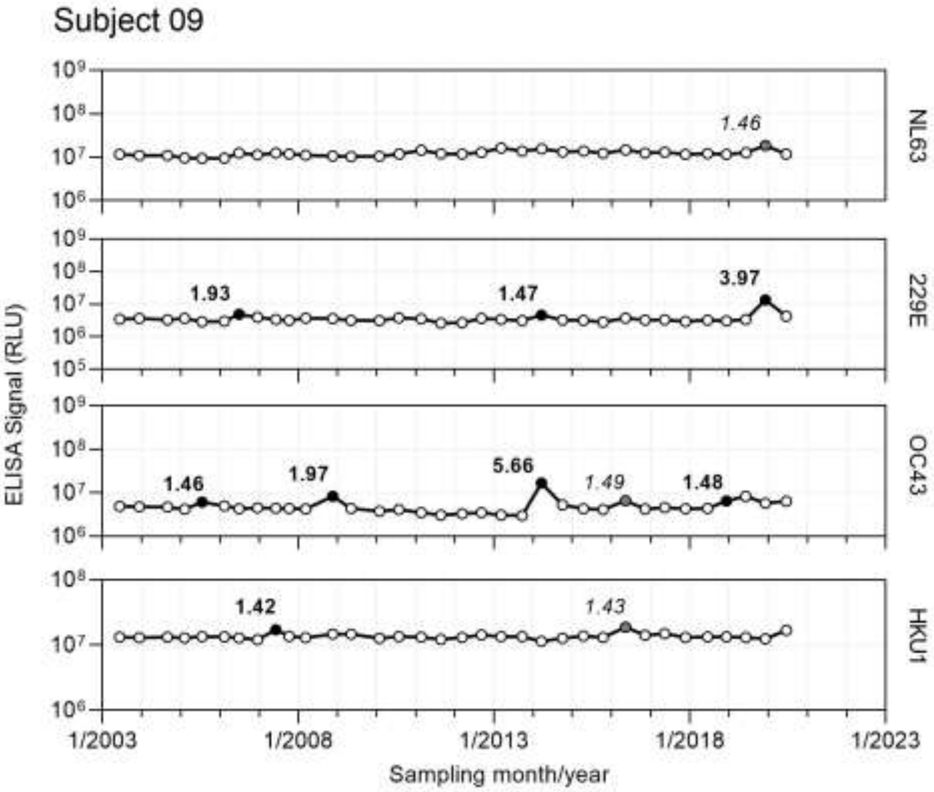

Supplementary Figure 1 (continued)

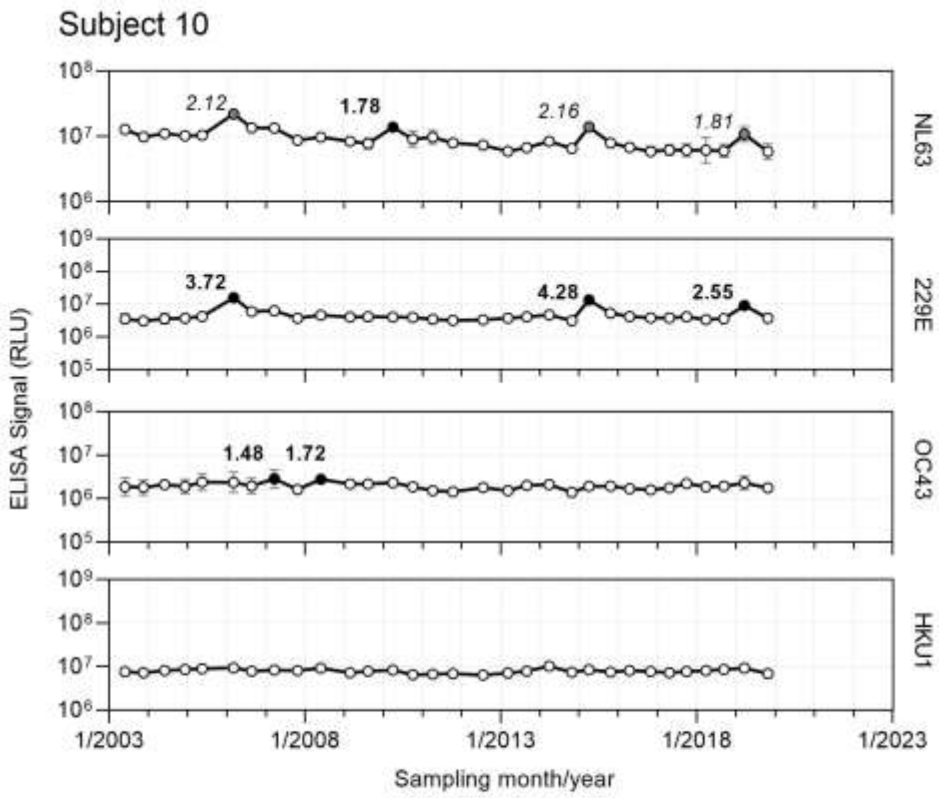

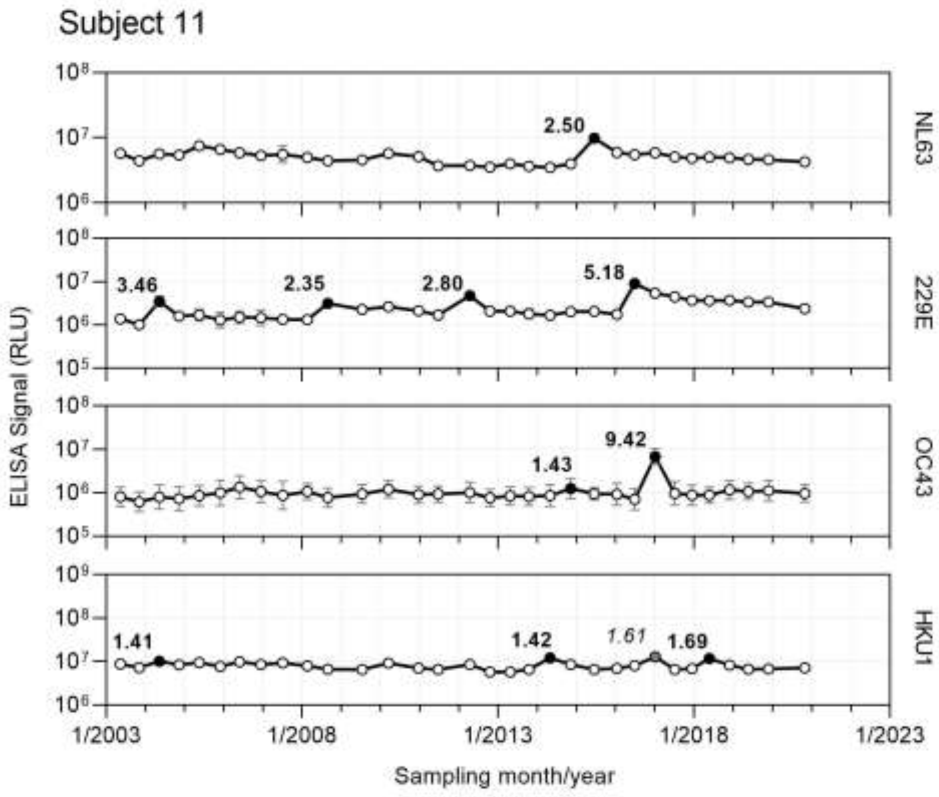

Supplementary Figure 1 (continued)

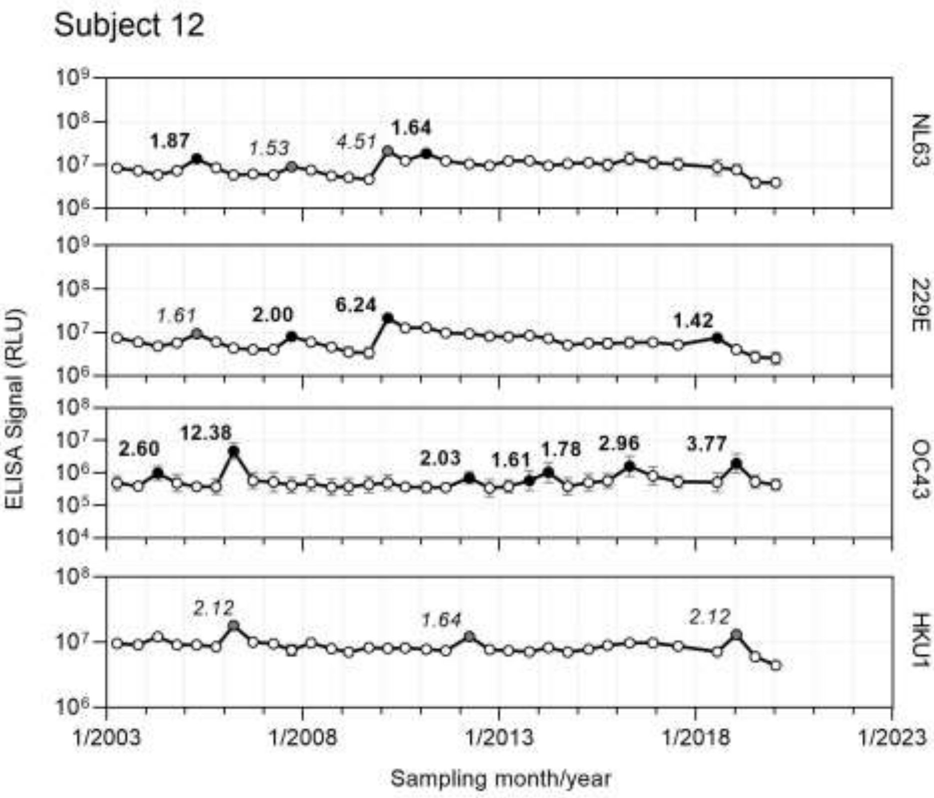

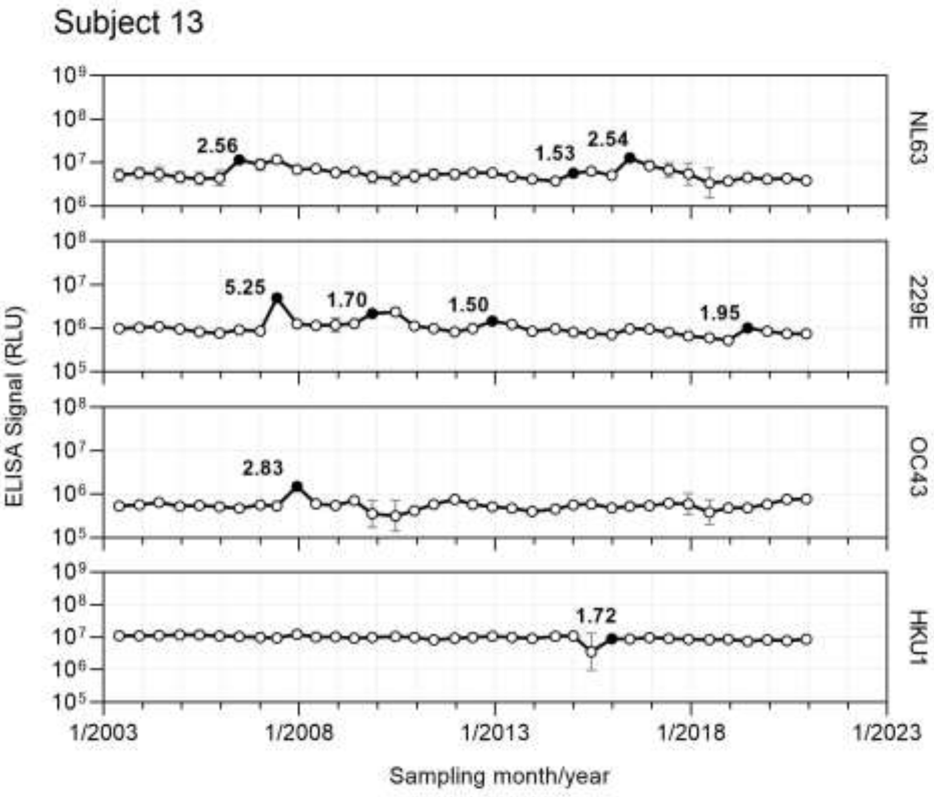

Supplementary Figure 1 (continued)

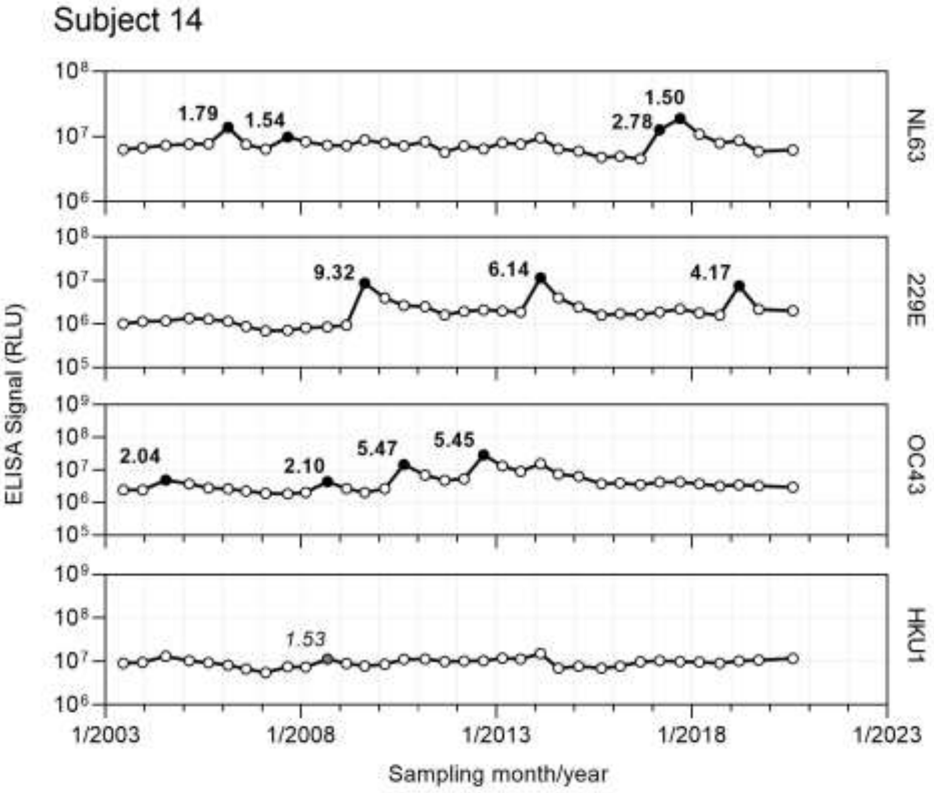

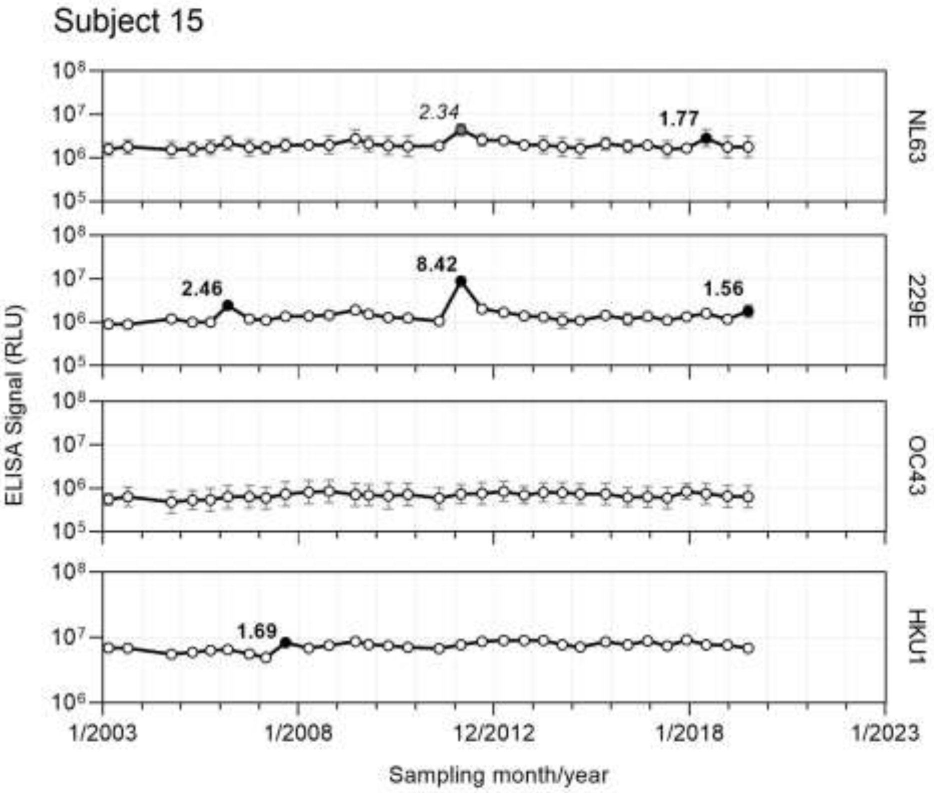

Supplementary Figure 1 (continued)

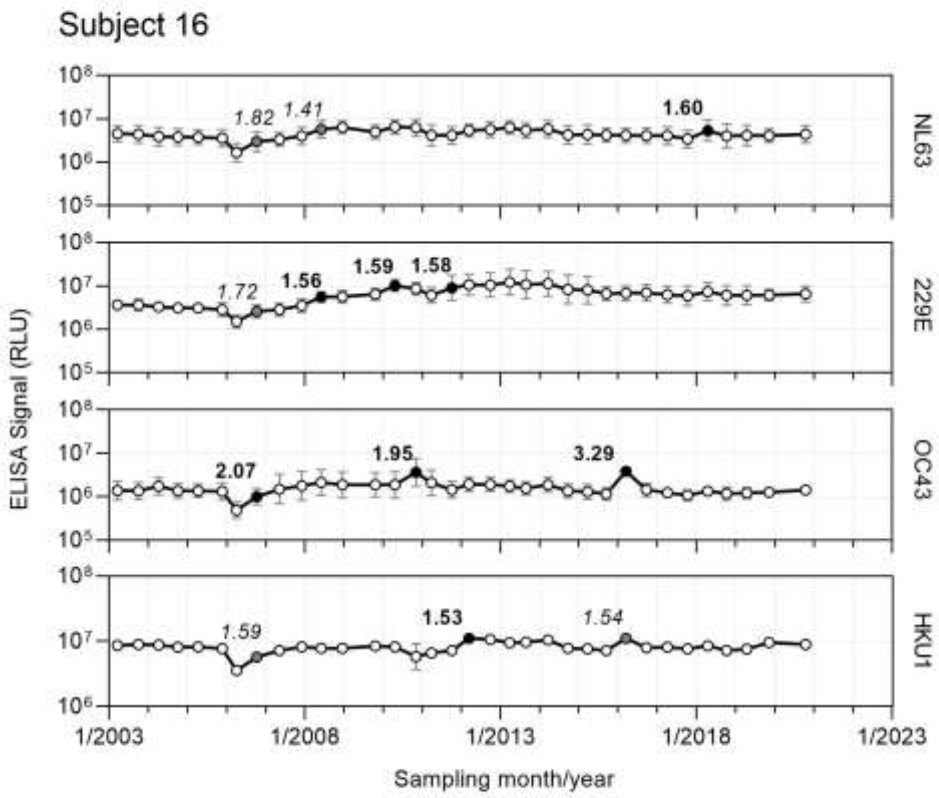

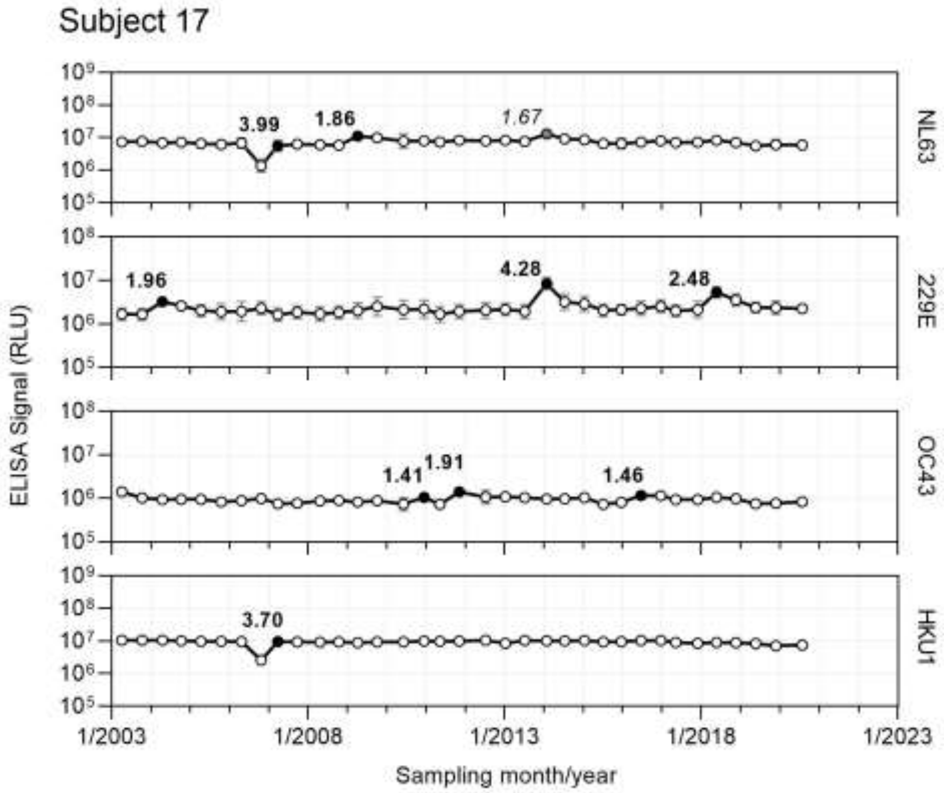

Supplementary Figure 1 (continued)

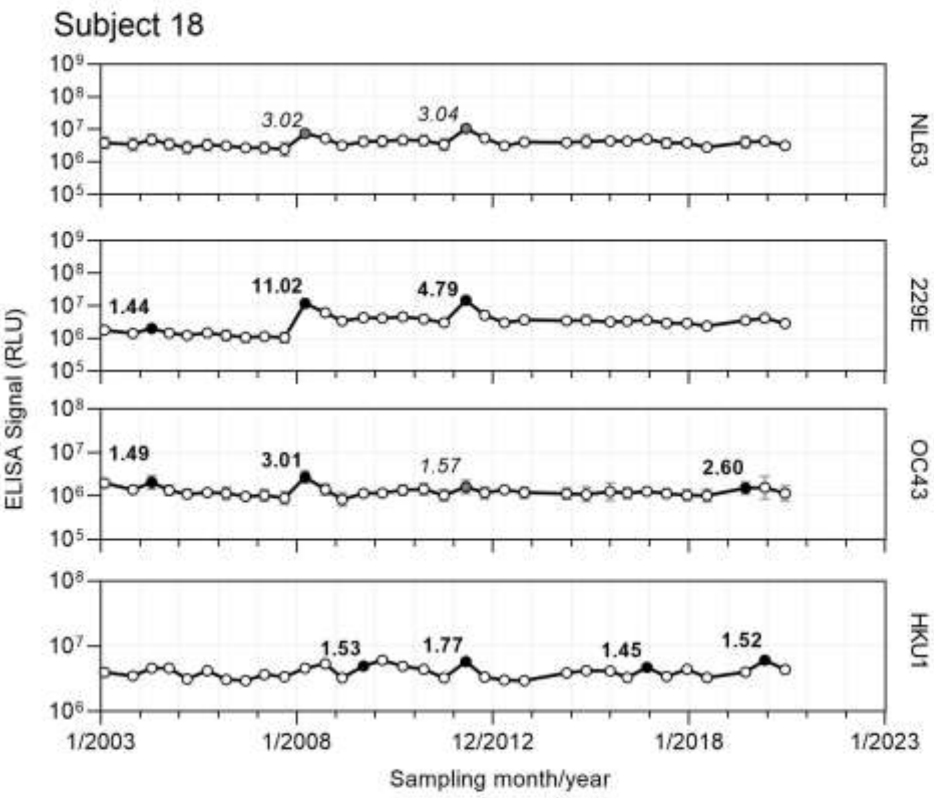

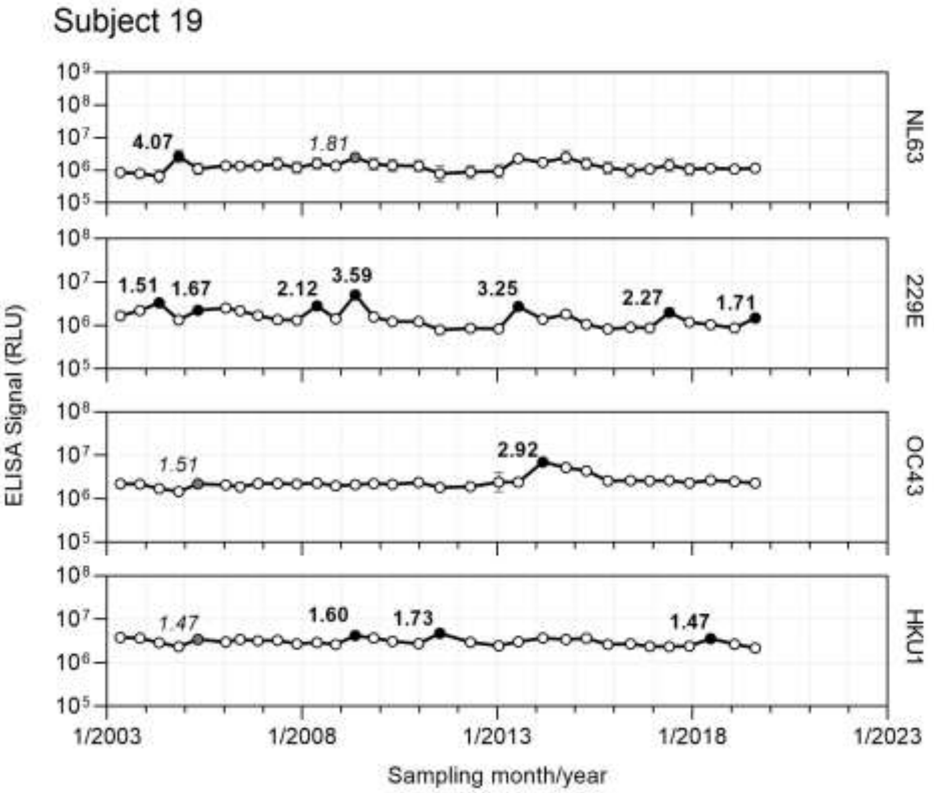

Supplementary Figure 1 (continued)

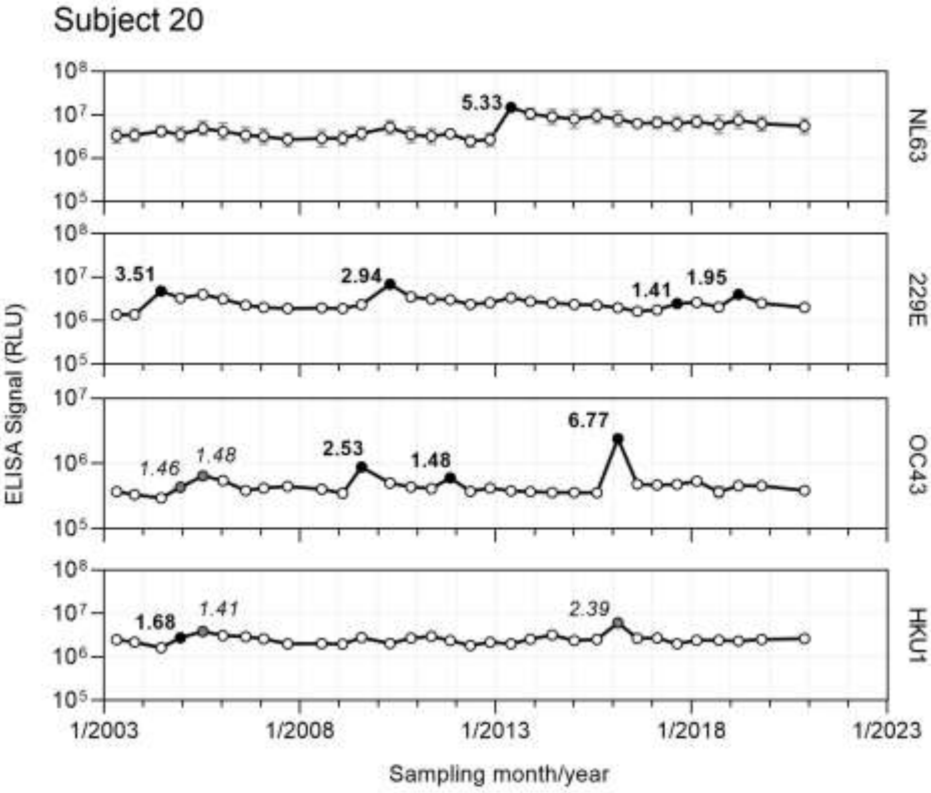

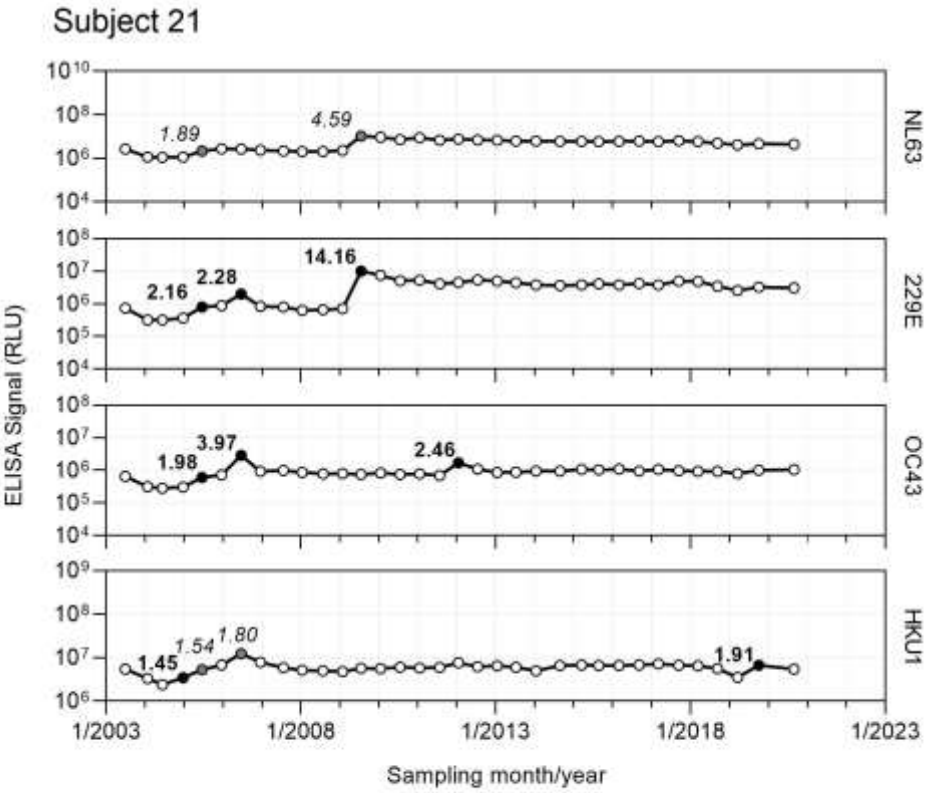

Supplementary Figure 1 (continued)

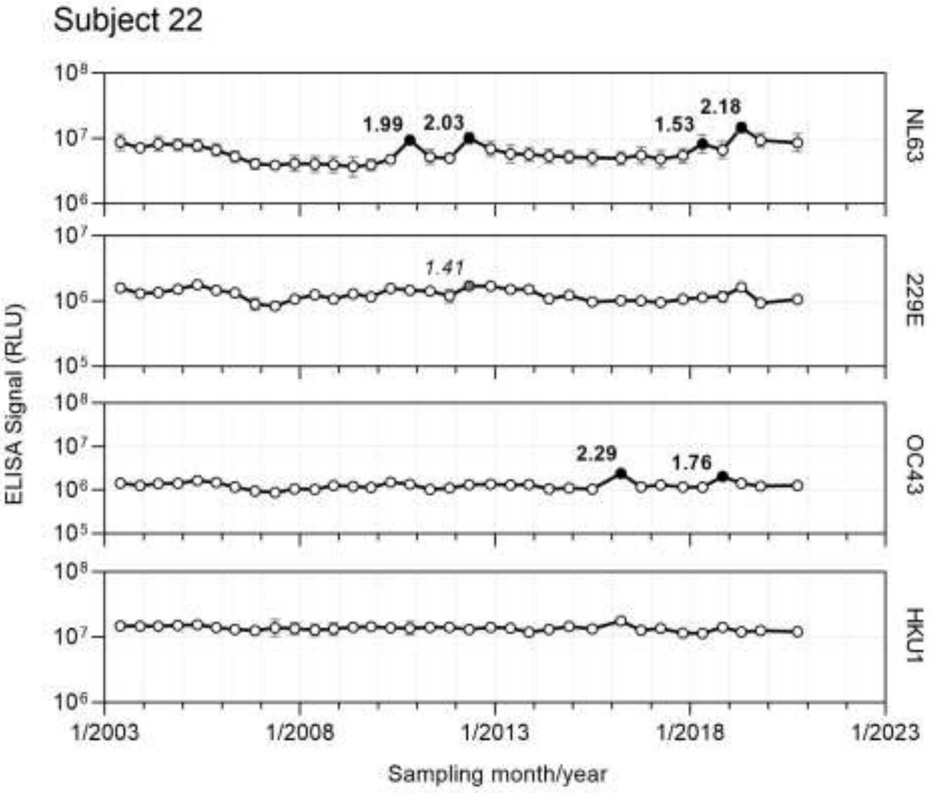

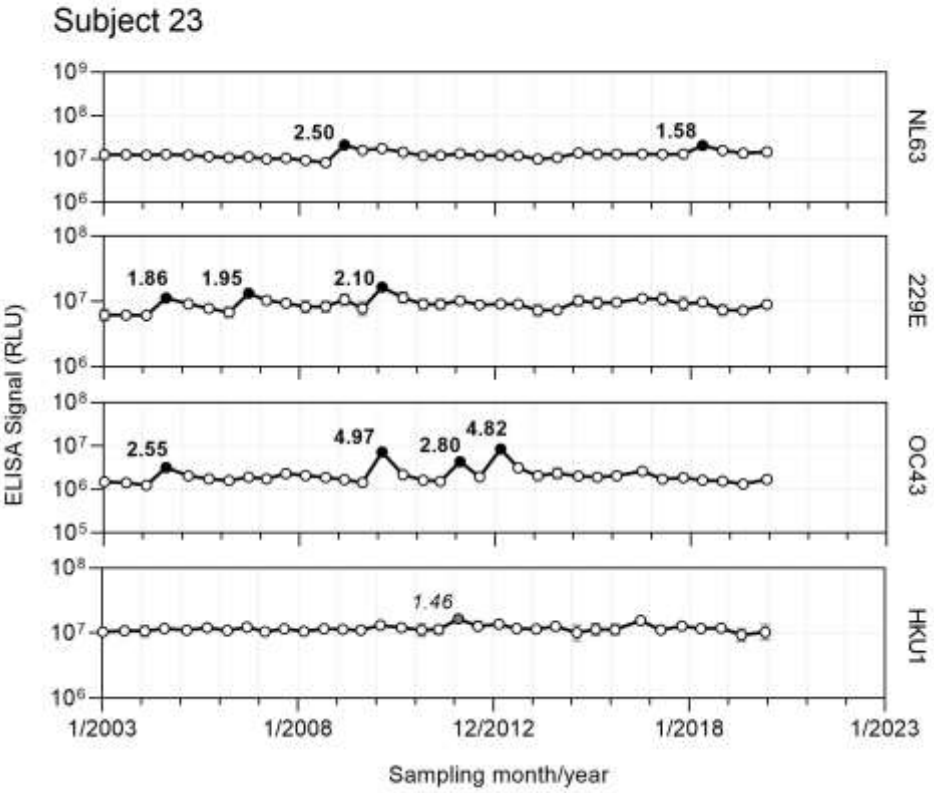

Supplementary Figure 1 (continued)

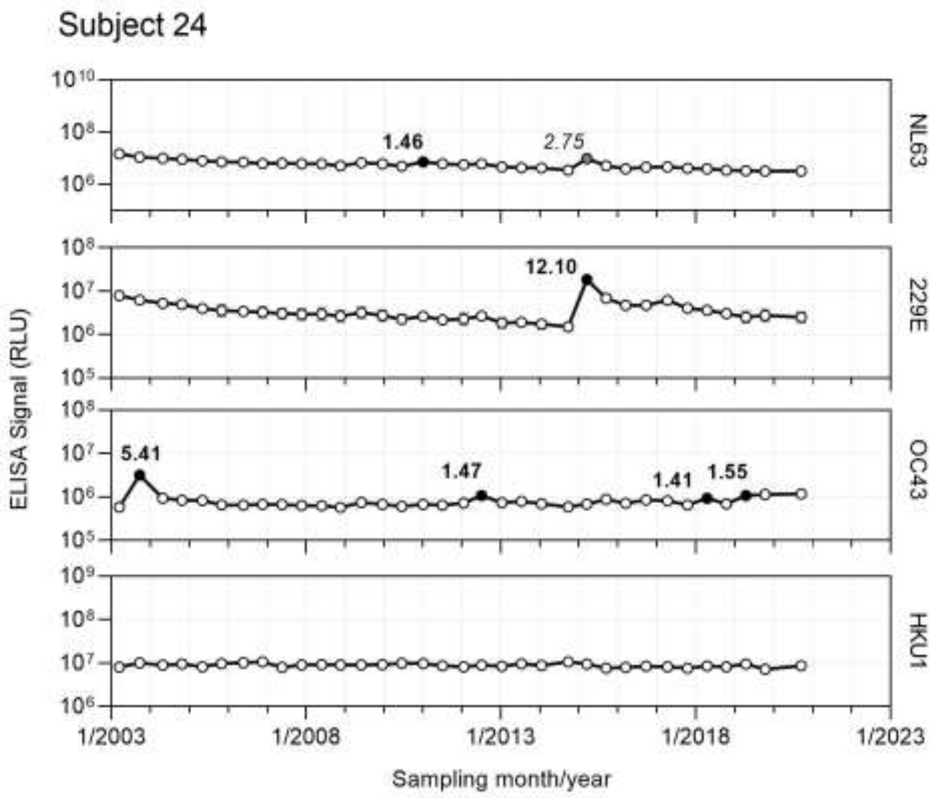

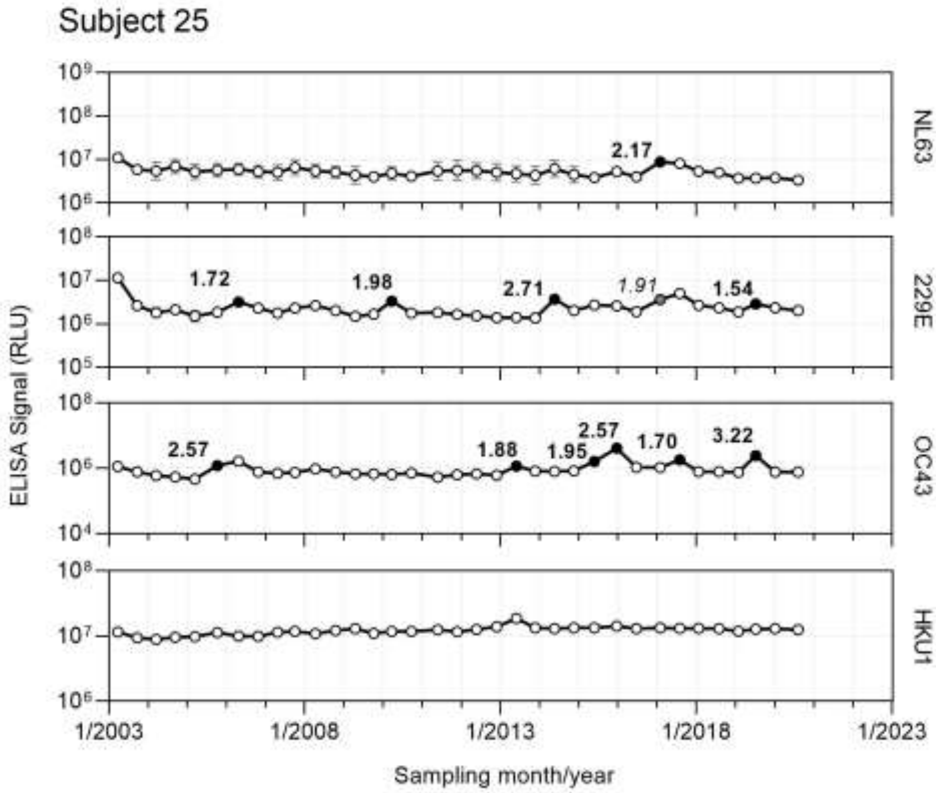

Supplementary Figure 1 (continued)

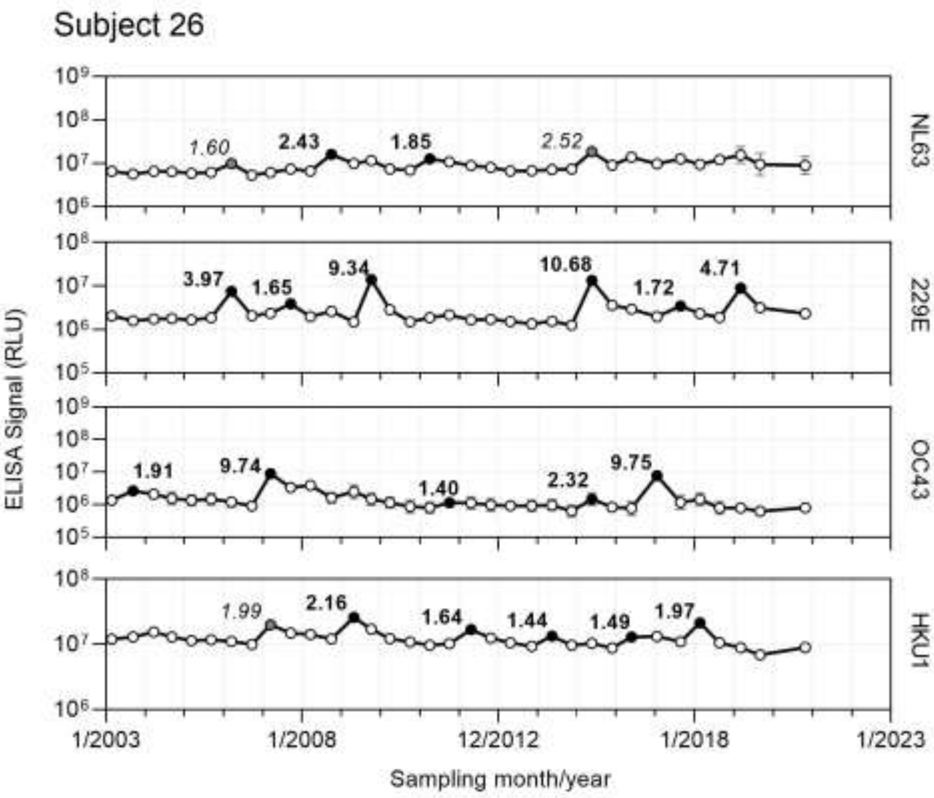

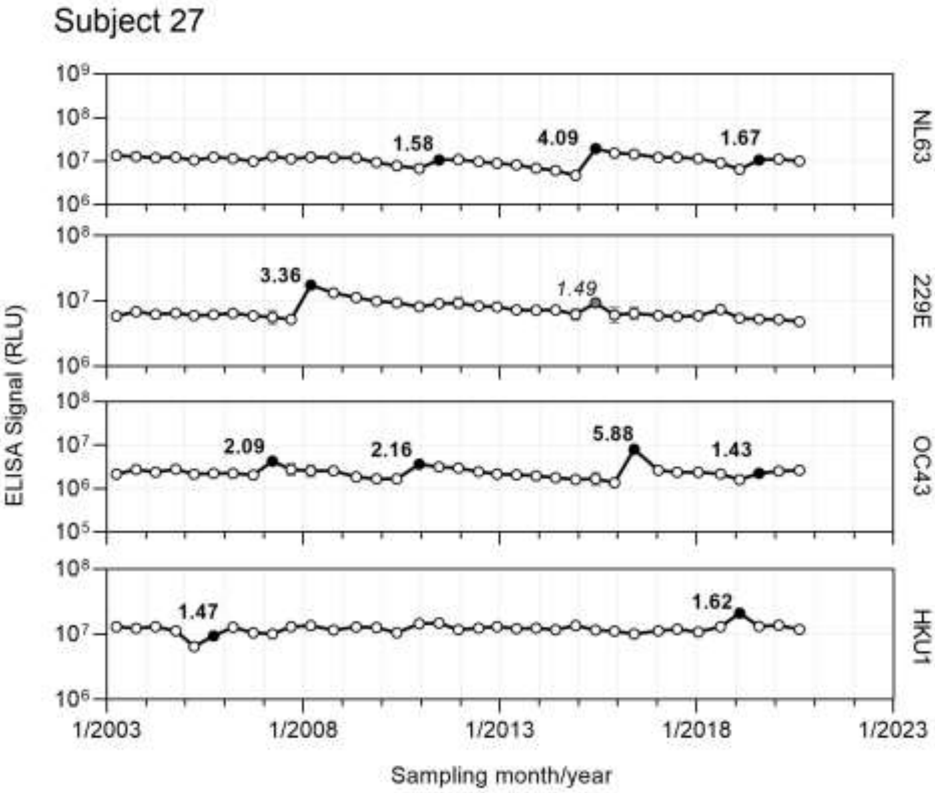

Supplementary Figure 1 (continued)

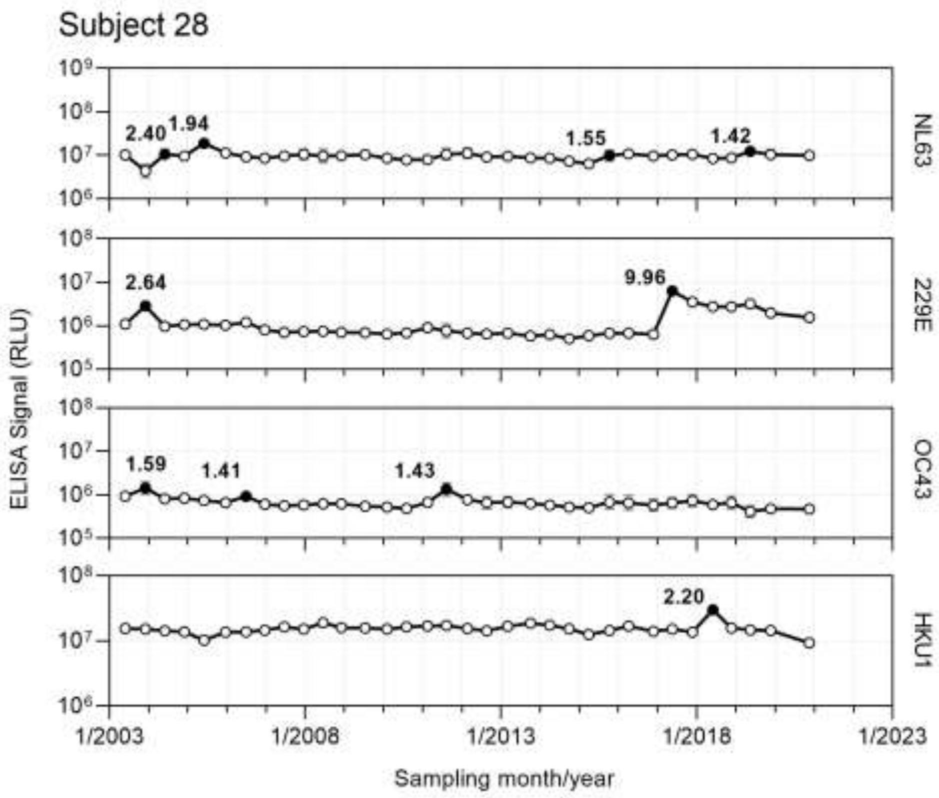

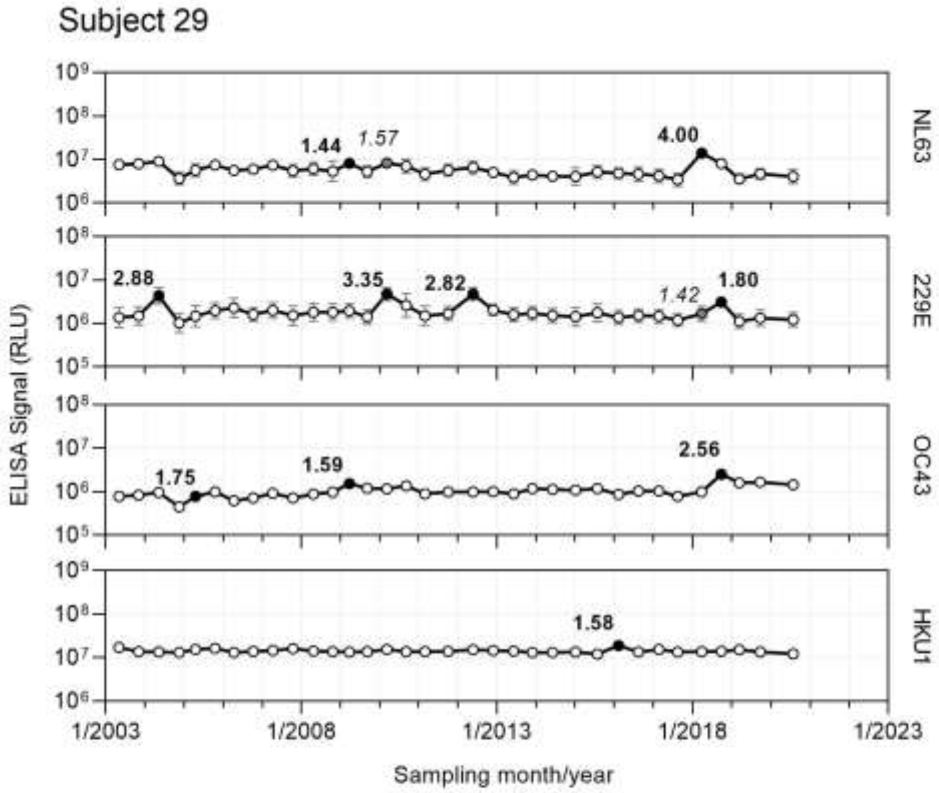

Supplementary Figure 1 (continued)

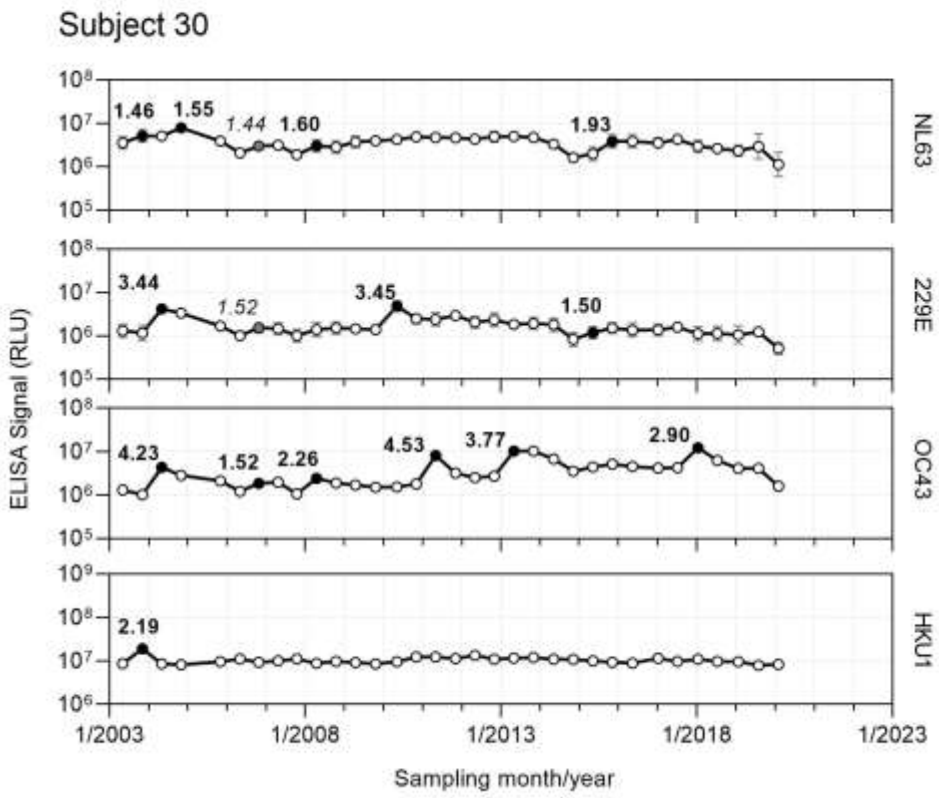

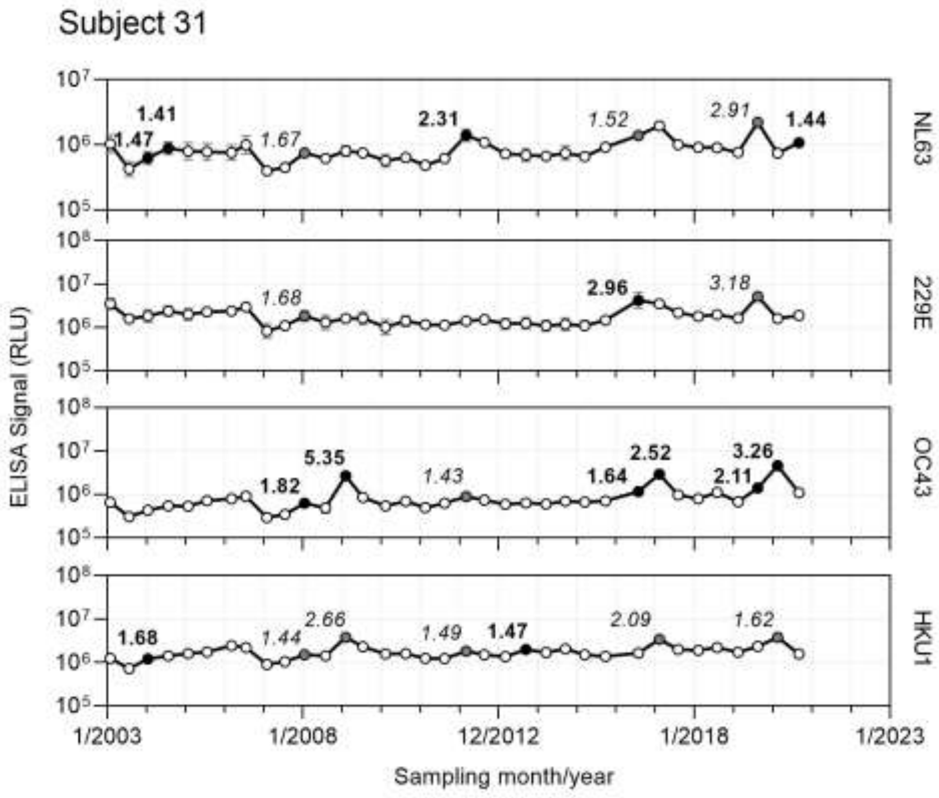

Supplementary Figure 1 (continued)

Subject 32

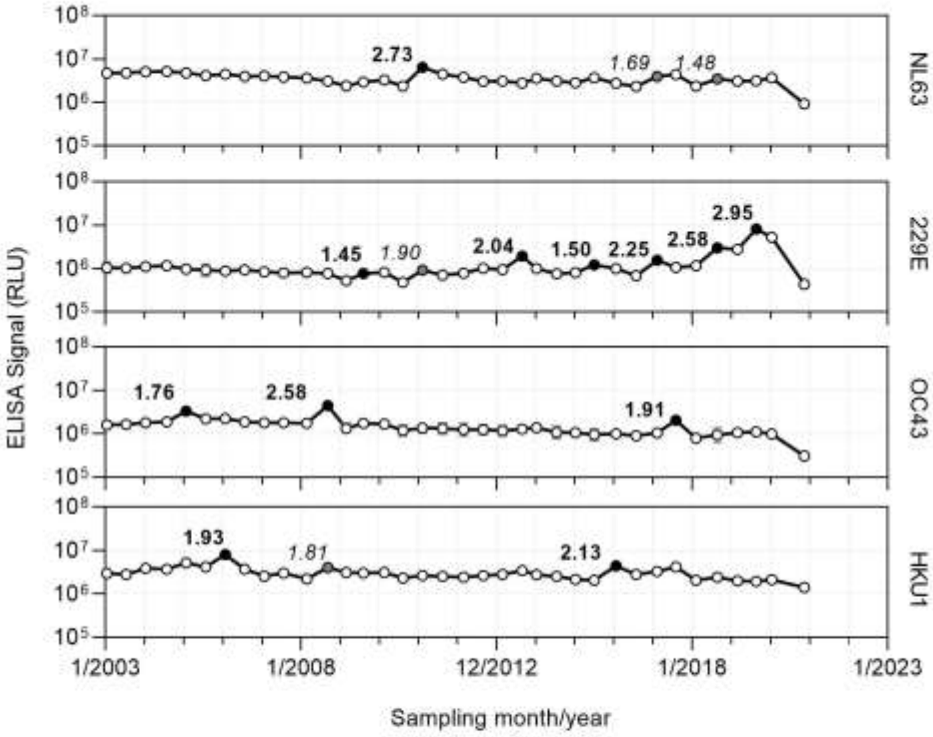

Subject 33

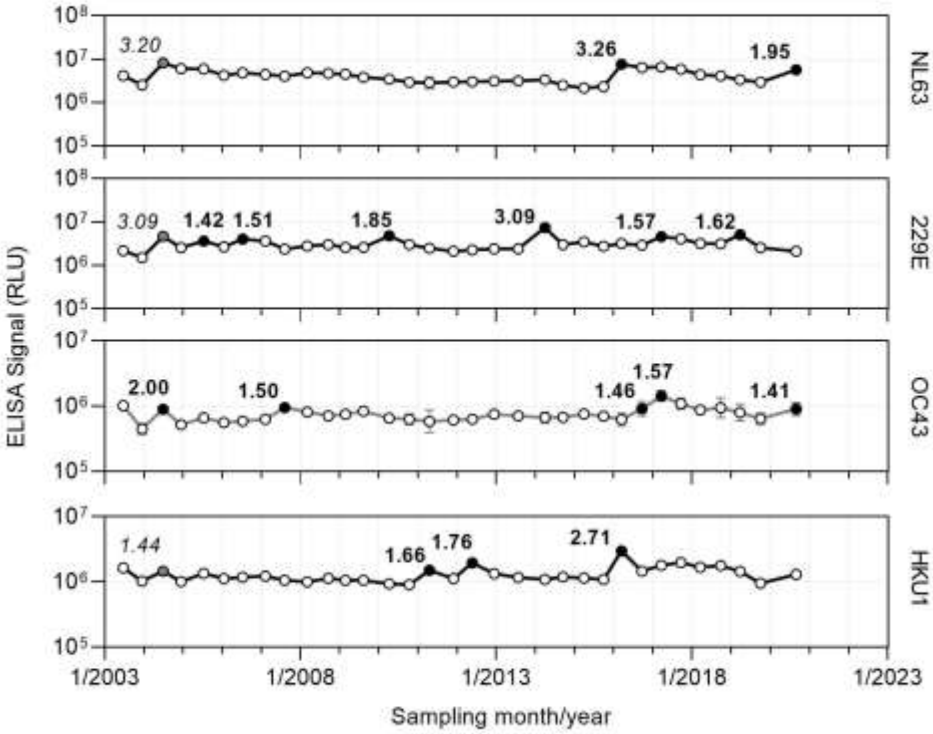

Supplementary Figure 1 (continued)

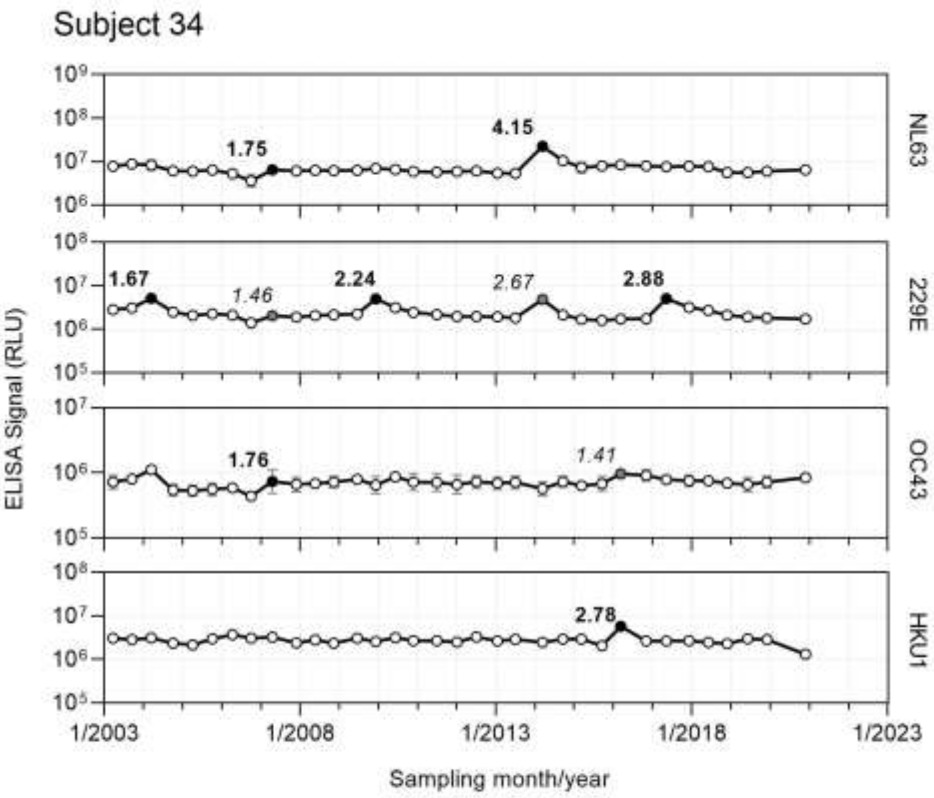

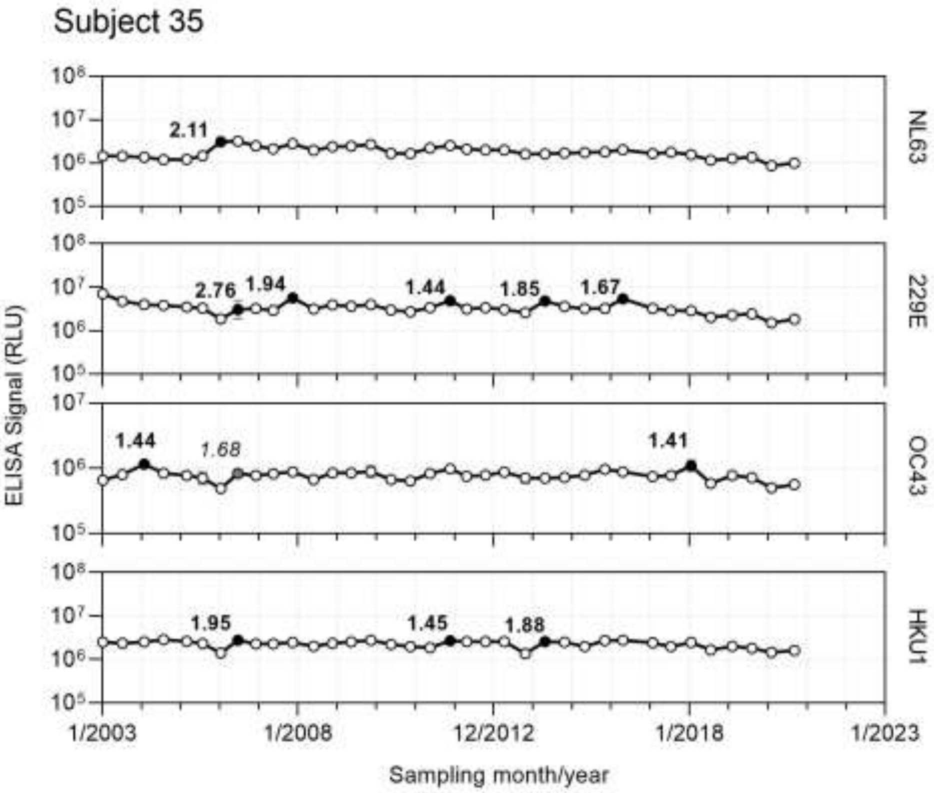

Supplementary Figure 1 (continued)

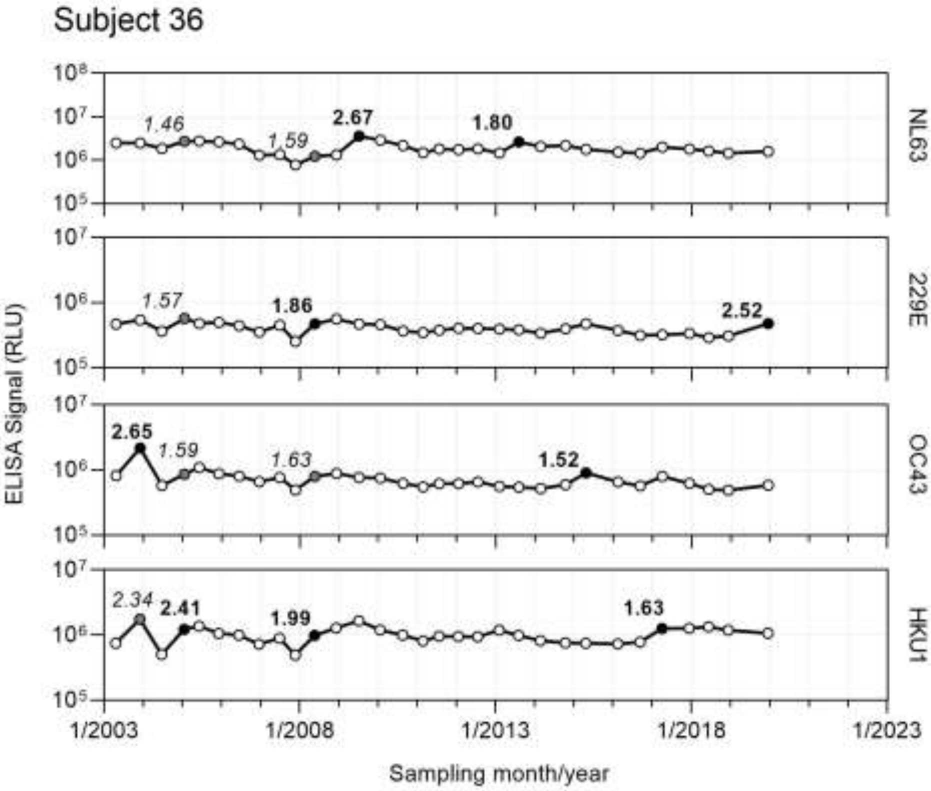

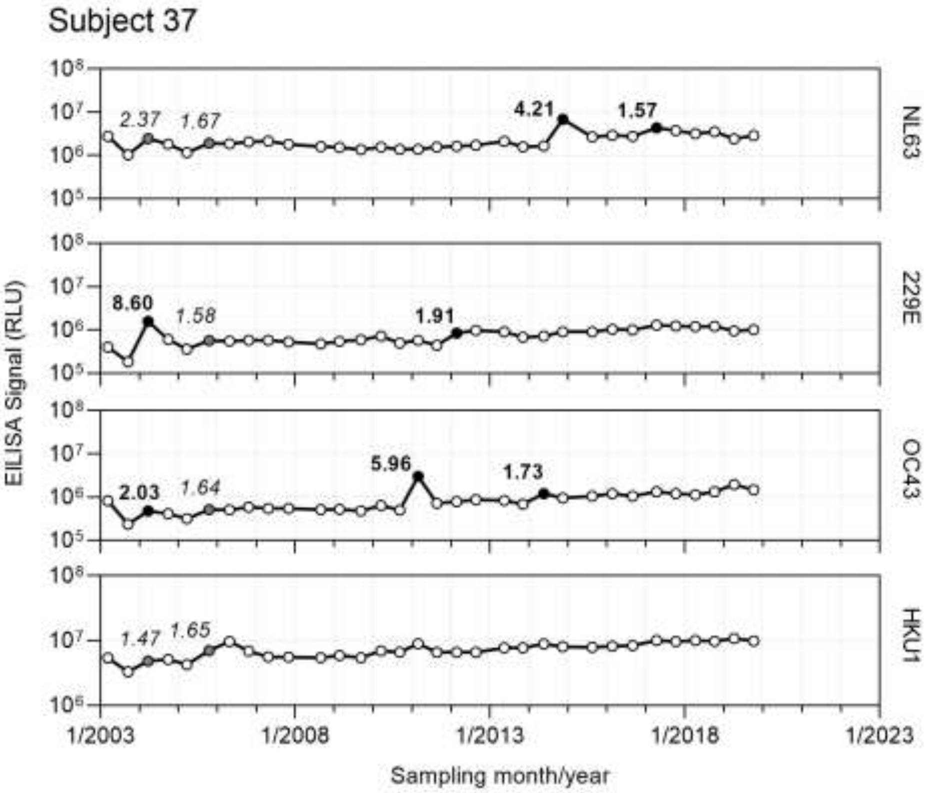

Supplementary Figure 1 (continued)

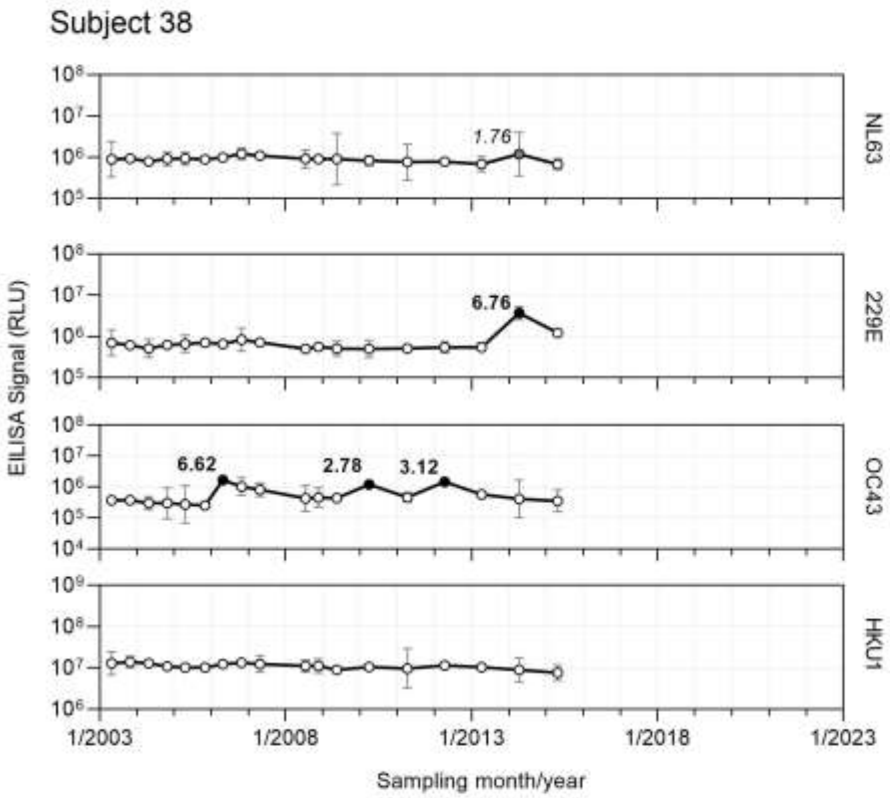

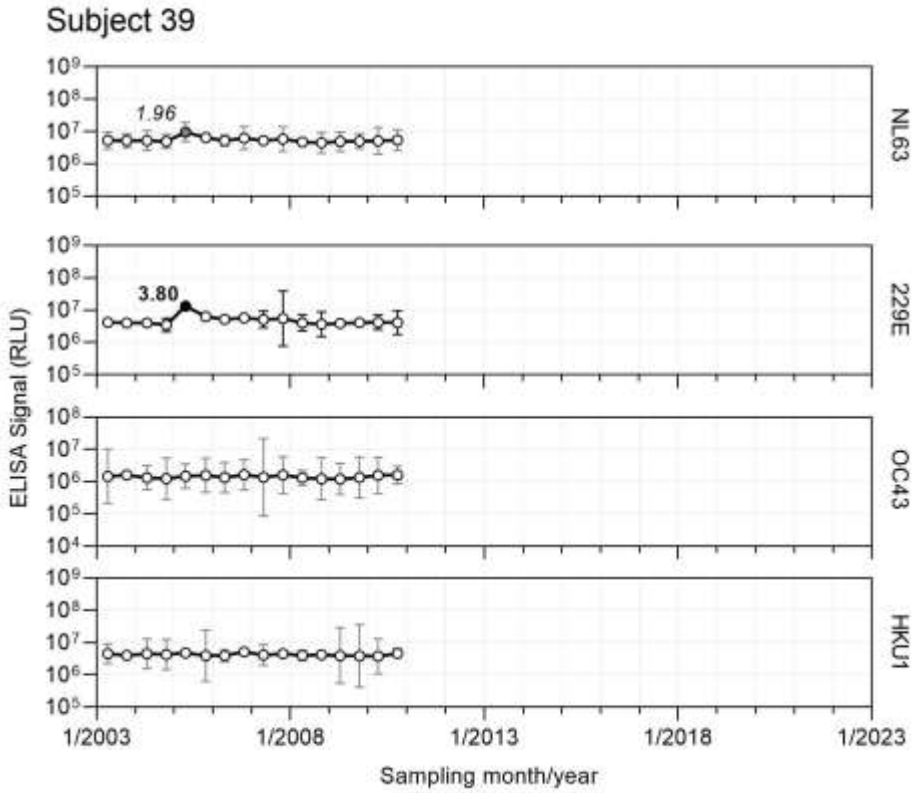

Supplementary Figure 1 (continued)

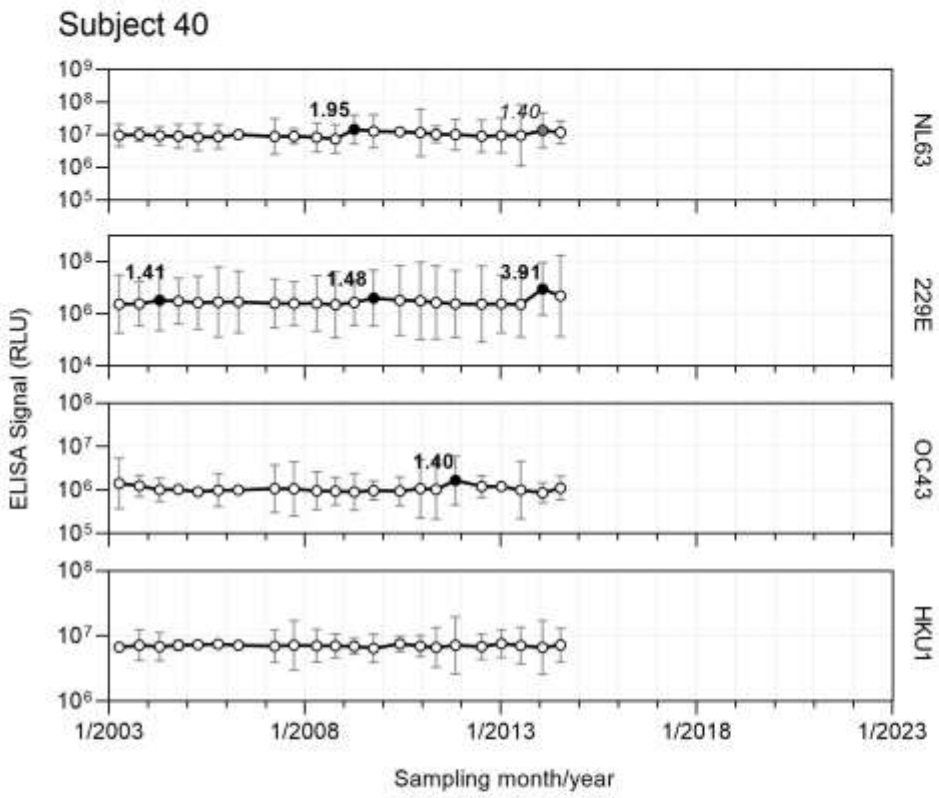

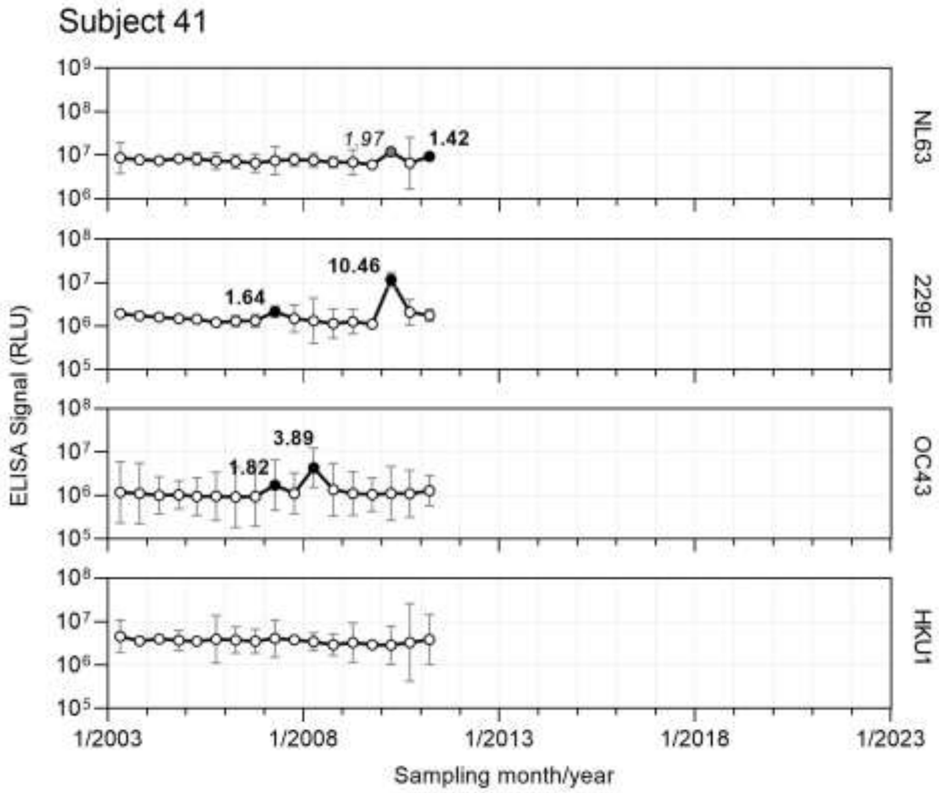

Supplementary Figure 1 (continued)

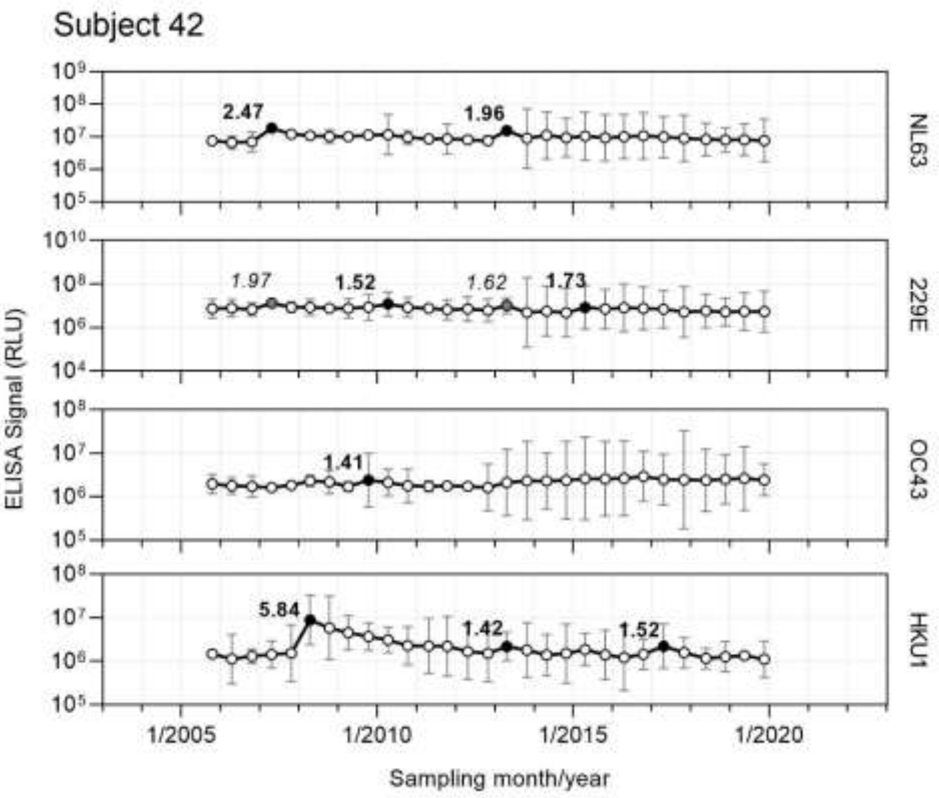

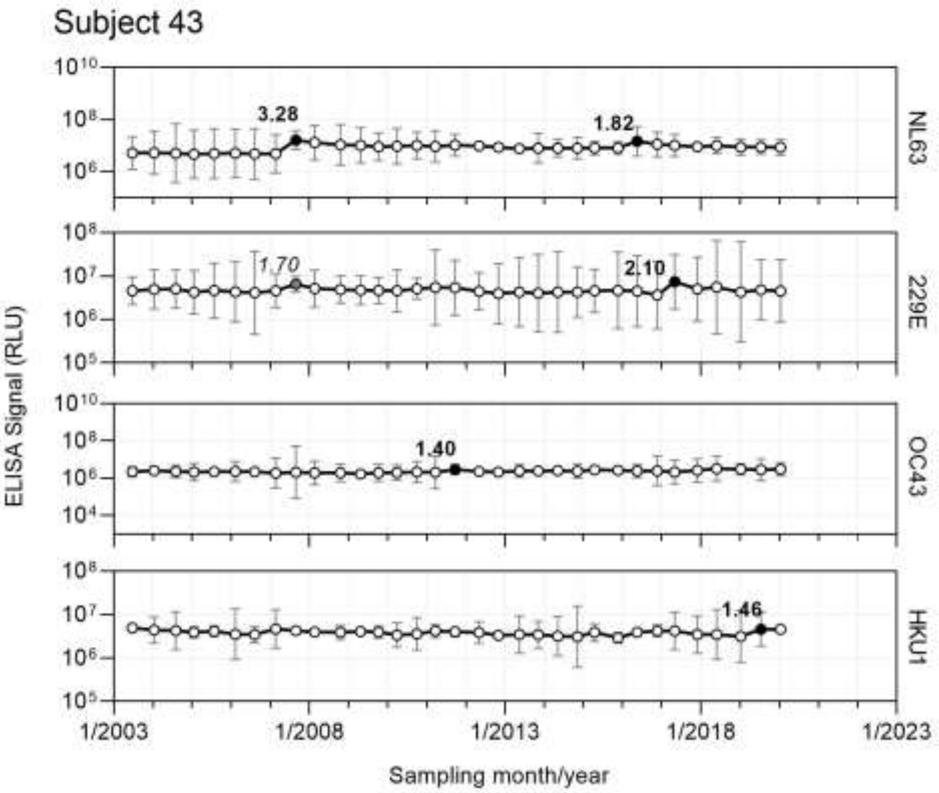

Supplementary Figure 1 (continued)

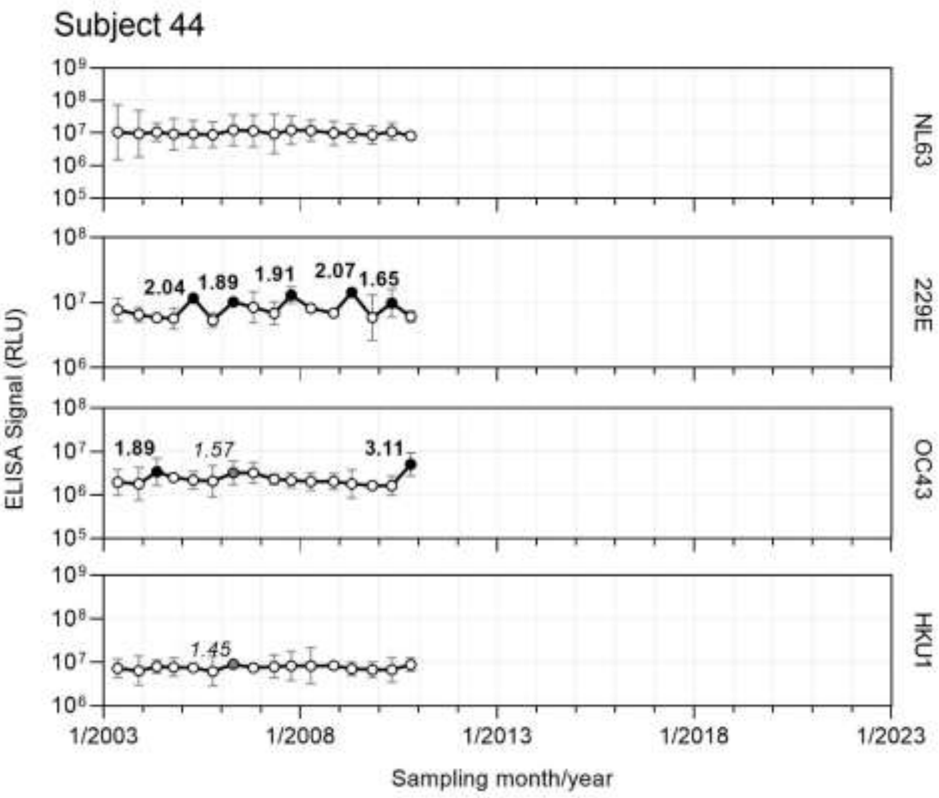

Supplementary Figure 2. Length comparison in years between HCoV reinfection interval with or without the presence of within-genus HCoV infection. *Each dot represents one reinfection interval. Vertical dashed line in all panels represent the median of all reinfection intervals. Vertical solid lines represent the median of each data set.*

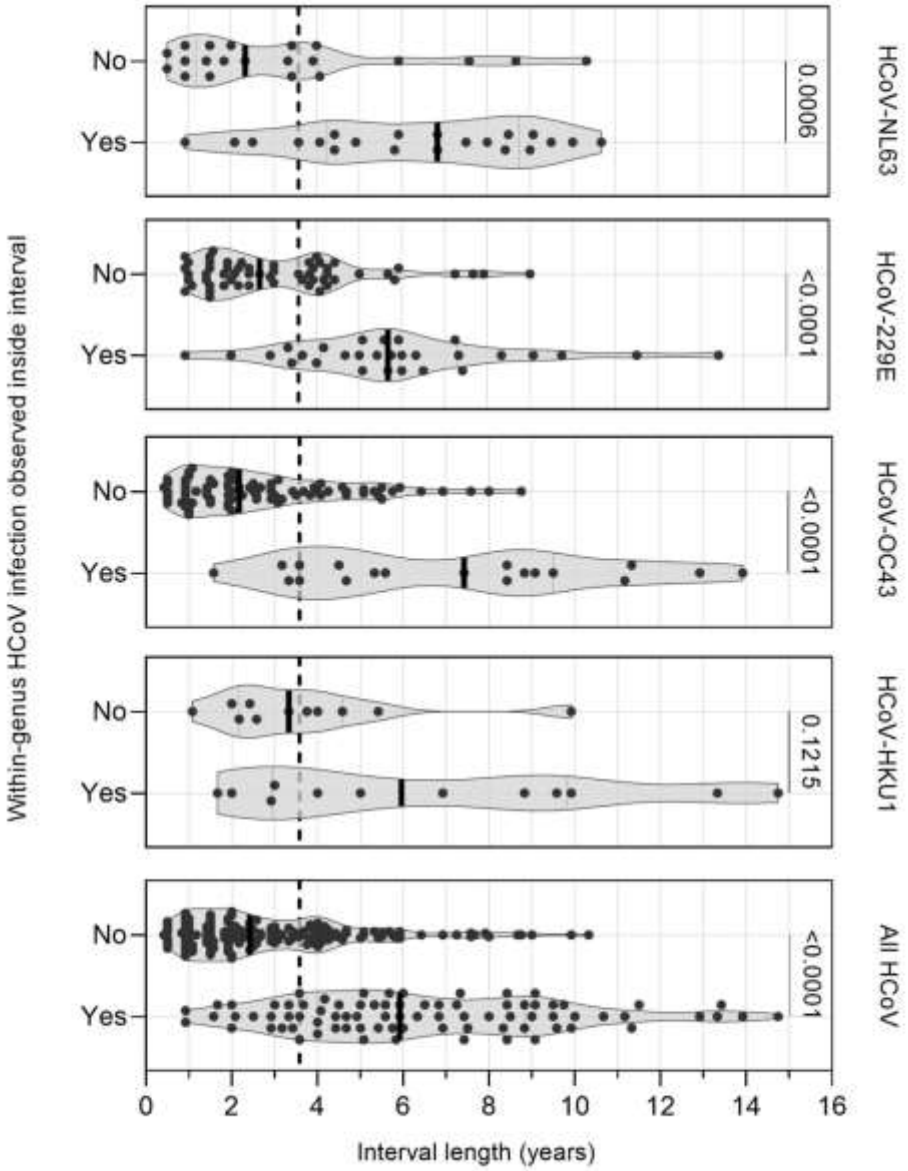

Supplement: Supplemental figures — Fig. S1 and S2. [file spectrum.03912-23-s0001.pdf]
