# Supplementary material for: Influenza-like illness symptoms due to endemic human coronavirus reinfections are not influenced by the length of the interval separating reinfections
Source: Microbiol Spectr. 2024 Feb 8;12(3):e03912-23. doi: 10.1128/spectrum.03912-23 (PMC10913438; doi:10.1128/spectrum.03912-23)
Supplement: Supplemental text and tables — Supplemental materials and methods and Tables S1 to S5. [file spectrum.03912-23-s0002.docx]

**Supplementary Material**

**Influenza like illness symptoms due to endemic human coronavirus reinfections are not influenced by the length of the interval separating reinfections**

Ferdyansyah Sechan^1,2^, Arthur W. D. Edridge^1,2^, Jacqueline van Rijswijk^1,2^, Maarten F. Jebbink^1,2^, Martin Deijs^1,2^, Margreet Bakker^1,2^, Amy Matser^2,3,4^, Maria Prins^2,5,6^, Lia van der Hoek*^1,2^

1 Laboratory of Experimental Virology, Department of Medical Microbiology and Infection Prevention, Amsterdam UMC, University of Amsterdam, Amsterdam, Netherlands.

2 Amsterdam Institute for Infection and Immunity, Amsterdam, Netherlands.

3 Amsterdam Public Health, Amsterdam, Netherlands

4 Netherlands Institute for Health Services Research (NIVEL), Utrecht, the Netherlands

5 Department of Infectious Diseases, Amsterdam UMC, University of Amsterdam, Amsterdam, the Netherlands

6 Department of Infectious Diseases, Public Health Service of Amsterdam, Amsterdam, the Netherlands

***Correspondence:** Lia van der Hoek ([c.m.vanderhoek@amsterdamumc.nl](mailto:c.m.vanderhoek@amsterdamumc.nl))

This file includes

- Specificity determination of partial HCoV-HKU1 nucleocapsid ELISA
- Supplementary Table 1-5

**Specificity determination of partial HCoV-HKU1 nucleocapsid ELISA**

We evaluated the possible cross-reactivity of HKU1-NLCt antigen with HCoV-OC43 antibodies by conducting ELISA on the serum samples of people aged 18 and older with PCR-confirmed infection by HCoV-OC43 (n = 13). These people were participants of the GRACE (Genomics to combat resistance against antibiotics in community-acquired LRTI in Europe) study, and they were enrolled due to either showing symptoms of acute lower respiratory infection or being diagnosed as such by their general practitioner (GP) [1]. Each subject provided two serum samples, one taken at the beginning of the study (V1) and the other after a month since the first visit (V2). We did the ELISA using the HKU1-NLCt antigen as previously described [2] on the V1 and V2 serum samples of HCoV-OC43-positive subjects. The antibody dynamic was represented by the fold-change value, or the ratio of ELISA signal at V2 and V1. We implemented the cut-off value of 1.40 to indicate infection (significant antibody rise); this cut-off value has been previously established [3]. We then compared the fold-change values of HKU1-NLCt assay on OC43-positive GRACE subjects with the corresponding values of OC43-NCt assay on these subjects. A cut-off of 10% was established previously for the difference between fold-change values of two HCoVs of the same genera (in this case, between HCoV-OC43 and HCoV-HKU1) [3]. When the difference is larger than 10%, the larger value is classified as the infection while the smaller one is designed as the cross-reaction [3].

The GRACE study was approved by the local ethics committees in all participating centers and by the competent authorities in each country: Cardiff and Southampton (United Kingdom): Southampton & South West Hampshire Research Ethics Committee A; Utrecht (Netherlands): Medisch Etische Toetsingcommissie Universitair Medisch Centrum Utrecht; Barcelona (Spain): Comité étic d’Investigació clínica Hospital Clínic de Barcelona; Mataro (Spain): Comitè d’Ètica d’Investigació Clínica (CEIC) del Consirci Sanitari del Maresme; Rotenburg (Germany): Ethik-Komission der Medizinischen Fakultät der Georg-August-Universitat Göttingen; Antwerp (Belgium): UZ Antwerpen Comité voor Medische Ethiek; Lodz, Szeczecin and Bialystok (Poland): Komisja Bioetyki Uniwersytetu Medycznego W Lodzi; Milano (Italy):IRCCS Fondazione Cà Granda Policlinico; Jonkoping (Sweden): Regionala etikprövningsnämden I Linköping; Bratislava (Slovakia): Etika Komisia Bratislavskeho; Gent (Belgium): Ethisch Comité Universitair Ziekenhuis Gent; Nice (France): Comité de Protection des Personnes Sud-Méditerranée II, Hôpital Salvator; and Jesenice (Slovenia): Komisija Republike Slovenije za Medicinsko Etiko.

Six out of thirteen (46%) HCoV-OC43-positive subjects showed the fold change value of ≥ 1.40 when assayed with the HKU1-NLCt ELISA. However, the actual fold-change values by either of HKU1-antigens were lower than fold-change by OC43-NCt assay, and all of them differed by more than 10% (**Supplementary Table 1**, values in bold). The low fold-change values by HKU1-NLCt implied that the cross-reactivity of this antigen was within a reasonable limit. Curiously, one OC43-positive subject showed an HKU1-NLCt assay fold-change value above the cut-off, and this person had an OC43-assay fold-change value below the cut-off (**Supplementary Table 1**, values underlined). It could be that this person had antibodies that recognize the L domain of HKU1-NLCt antigen, which shares a high per cent similarity with the L domain of HCoV-OC43 N. Since HKU1-NCt has not been shown to cross-react with antibodies against HCoV-NL63 and HCoV-229E [3], as well as the HKU1-NLCt antigen having less than 25% identity with the corresponding amino acid sequence in these HCoVs, we deemed such cross-reactivity to be highly unlikely and did not test for them using HKU1-NLCt antigen.

We checked whether HKU1-NLCt might also react to antibodies against non-HCoVs by running the ELISA on V1 and V2 serum samples from 24 subjects from the GRACE Study with no HCoV infection but infected with other respiratory viruses. These respiratory viruses (and the number of subjects infected by them in parentheses) were Bocavirus (1), Adenovirus (1), Parainfluenza virus (2), Respiratory Syncytial Virus (6), influenza virus (7), and human rhinovirus (7). ELISA signal fold change was similarly calculated from ELISA signal value at V2 divided by V1, and 1.40 cut-off was again used for significant antibody increase [3]. Out of 24 subjects, only one showed a value above 1.40 (**Supplementary Table S2**, values in bold). This subject was confirmed positive for Bocavirus, and we observed a similarly high value with the HKU1-NCt assay. Therefore, we concluded that the HKU1-NLCt assay is quite specific (96%) against non-HCoVs.

**References:**

1. Ieven M, Coenen S, Loens K, et al. Aetiology of lower respiratory tract infection in adults in primary care: a prospective study in 11 European countries. Clinical Microbiology and Infection [Internet]. Elsevier Ltd; **2018**; 24(11):1158–1163. Available from: https://doi.org/10.1016/j.cmi.2018.02.004

2. Sechan F, Grobben M, Edridge AWD, et al. Atypical Antibody Dynamics During Human Coronavirus HKU1 Infections. Front Microbiol [Internet]. **2022**; 13. Available from: https://www.frontiersin.org/articles/10.3389/fmicb.2022.853410/full

3. Edridge AWD, Kaczorowska J, Hoste ACR, et al. Seasonal coronavirus protective immunity is short-lasting. Nat Med [Internet]. Springer US; **2020**; 26(11):1691–1693. Available from: http://dx.doi.org/10.1038/s41591-020-1083-1

**Supplementary Table 1** Raw ELISA signal values and fold change of HCoV-OC43-infected subjects from the GRACE study tested with HKU1-NLCt antigen and OC43-NCT antigen. Bold values indicate significant increase (≥ 1.40) for both HKU1-NLCt- and OC43-NCt-assay. Underlined values indicate significant increase (≥ 1.40) for HKU1-NLCt-assay but not OC43-NCt-assay.

| **ID** | **HKU1-NLCt (RLU)** | | **OC43-NCt (RLU)** | | **Fold-change HKU1-NLCt** | **Fold-change OC43-NCt** |
| --- | --- | --- | --- | --- | --- | --- |
|  | **V1** | **V2** | **V1** | **V2** |  |  |
| C4091 | 2.01E+06 | 4.89E+06 | 9.54E+05 | 1.26E+07 | **2.43** | **13.24** |
| J1340 | 1.92E+06 | 2.63E+06 | 1.46E+06 | 1.45E+07 | 1.37 | 9.99 |
| S2817 | 4.39E+06 | 4.91E+06 | 1.86E+06 | 4.81E+06 | 1.12 | 2.58 |
| S2719 | 3.19E+06 | 1.11E+07 | 1.32E+06 | 1.18E+07 | **3.46** | **8.93** |
| R0730 | 3.87E+06 | 4.24E+06 | 2.62E+06 | 2.72E+06 | 1.10 | 1.04 |
| Q0239 | 3.01E+06 | 4.17E+06 | 1.40E+06 | 9.87E+06 | **1.39** | **7.07** |
| S2733 | 6.20E+06 | 9.63E+06 | 1.28E+06 | 1.51E+06 | 1.55 | 1.18 |
| I4965 | 3.52E+06 | 7.08E+06 | 1.47E+06 | 1.36E+07 | **2.01** | **9.19** |
| S2705 | 2.72E+06 | 4.79E+06 | 1.46E+06 | 1.88E+07 | **1.76** | **12.89** |
| S2677 | 7.33E+05 | 7.71E+05 | 1.14E+06 | 1.15E+06 | 1.05 | 1.02 |
| S2551 | 6.85E+06 | 7.42E+06 | 2.77E+06 | 1.31E+07 | 1.08 | 4.71 |
| O2645 | 2.33E+06 | 2.96E+06 | 1.49E+06 | 1.61E+06 | 1.27 | 1.08 |
| Q0491 | 2.28E+06 | 5.38E+06 | 2.40E+06 | 1.66E+07 | **2.37** | **6.90** |

**Supplementary Table 2** Raw ELISA signal values and fold change of GRACE study subjects infected by viruses other than HCoVs as tested with HKU1-NLCt and HKU1 NCt antigen. Bold values indicate significant increase (≥ 1.40) for both assays.

| **ID** | **HKU1-NLCt (RLU)** | | **HKU1-NCt (RLU)** | | **Fold-change HKU1-NLCt** | **Fold-change HKU1-NCt** |
| --- | --- | --- | --- | --- | --- | --- |
|  | **V1** | **V2** | **V1** | **V2** |  |  |
| P0812 | 2.48E+06 | 4.76E+06 | 1.90E+06 | 2.82E+06 | **1.92** | **1.48** |
| O2757 | 3.63E+06 | 2.58E+06 | 6.34E+05 | 7.26E+05 | 0.71 | 1.15 |
| Q1261 | 3.62E+06 | 3.10E+06 | 2.16E+06 | 2.01E+06 | 0.86 | 0.93 |
| H4992 | 2.17E+06 | 2.14E+06 | 1.35E+06 | 1.30E+06 | 0.99 | 0.96 |
| C4147 | 3.02E+06 | 3.17E+06 | 1.73E+06 | 1.84E+06 | 1.05 | 1.06 |
| D0158 | 5.33E+06 | 4.36E+06 | 3.46E+06 | 3.10E+06 | 0.82 | 0.90 |
| E4079 | 2.43E+06 | 2.13E+06 | 1.12E+06 | 1.10E+06 | 0.88 | 0.98 |
| I1591 | 2.06E+06 | 1.99E+06 | 1.08E+06 | 1.10E+06 | 0.96 | 1.02 |
| O0363 | 2.44E+06 | 2.46E+06 | 1.39E+06 | 1.46E+06 | 1.01 | 1.05 |
| B4412 | 2.80E+06 | 2.46E+06 | 2.42E+06 | 2.28E+06 | 0.88 | 0.94 |
| C3265 | 1.75E+06 | 1.78E+06 | 1.12E+06 | 1.09E+06 | 1.02 | 0.97 |
| C0129 | 4.36E+06 | 3.78E+06 | 2.63E+06 | 2.09E+06 | 0.87 | 0.80 |
| K0851 | 2.12E+06 | 1.95E+06 | 9.88E+05 | 9.99E+05 | 0.92 | 1.01 |
| I4839 | 1.21E+07 | 1.22E+07 | 8.71E+06 | 8.86E+06 | 1.01 | 1.02 |
| I0527 | 3.21E+06 | 3.68E+06 | 2.35E+06 | 4.07E+06 | 1.15 | 1.73 |
| S1669 | 4.21E+06 | 4.02E+06 | 1.90E+06 | 1.94E+06 | 0.96 | 1.02 |
| O0251 | 3.04E+06 | 2.87E+06 | 1.69E+06 | 1.61E+06 | 0.95 | 0.95 |
| H2332 | 1.48E+06 | 1.64E+06 | 6.86E+05 | 7.77E+05 | 1.11 | 1.13 |
| E4345 | 7.50E+06 | 5.86E+06 | 2.62E+06 | 2.41E+06 | 0.78 | 0.92 |
| C4651 | 7.67E+06 | 7.55E+06 | 5.22E+06 | 5.15E+06 | 0.99 | 0.99 |
| H0694 | 4.42E+06 | 4.39E+06 | 3.25E+06 | 3.46E+06 | 0.99 | 1.06 |
| B0926 | 2.50E+06 | 2.59E+06 | 1.49E+06 | 1.61E+06 | 1.03 | 1.08 |
| E1363 | 4.89E+06 | 3.47E+06 | 2.75E+06 | 1.98E+06 | 0.71 | 0.72 |
| F3212 | 6.91E+06 | 6.60E+06 | 3.34E+06 | 3.11E+06 | 0.96 | 0.93 |

**Supplementary Table 3** Duration of follow up in years, year and age at the start and end of follow up, and the frequency of infection for each and all endemic HCoV per subject.

| **Subject** | **Year start** | **Year end** | **Age start** | **Age end** | **Year duration** | **HCoV-NL63** | **HCoV-229E** | **HCoV-OC43** | **HCoV-HKU1** | **All HCoV** |
| --- | --- | --- | --- | --- | --- | --- | --- | --- | --- | --- |
| 1 | 2003 | 2020 | 31 | 49 | 17.50 | 2 | 3 | 6 | 1 | 10 |
| 2 | 2003 | 2020 | 31 | 49 | 17.50 | 3 | 2 | 9 | 1 | 14 |
| 3 | 2003 | 2020 | 36 | 53 | 17.33 | 3 | 3 | 7 | 1 | 12 |
| 4 | 2003 | 2019 | 35 | 52 | 16.83 | 4 | 2 | 2 | 1 | 8 |
| 5 | 2003 | 2019 | 27 | 43 | 15.92 | 2 | 3 | 1 | 0 | 6 |
| 6 | 2003 | 2019 | 23 | 39 | 16.50 | 2 | 4 | 4 | 0 | 8 |
| 7 | 2003 | 2020 | 26 | 43 | 17.42 | 0 | 2 | 4 | 1 | 7 |
| 8 | 2003 | 2019 | 26 | 43 | 16.58 | 3 | 3 | 5 | 1 | 11 |
| 9 | 2003 | 2019 | 28 | 45 | 16.42 | 0 | 3 | 4 | 1 | 7 |
| 10 | 2003 | 2019 | 31 | 48 | 16.33 | 1 | 3 | 2 | 0 | 6 |
| 11 | 2003 | 2019 | 30 | 46 | 16.50 | 1 | 4 | 2 | 3 | 9 |
| 12 | 2003 | 2020 | 34 | 50 | 16.75 | 2 | 3 | 7 | 0 | 12 |
| 13 | 2003 | 2020 | 33 | 51 | 17.50 | 3 | 4 | 1 | 1 | 9 |
| 14 | 2003 | 2020 | 35 | 52 | 17.08 | 4 | 3 | 4 | 0 | 11 |
| 15 | 2003 | 2019 | 27 | 43 | 16.33 | 1 | 3 | 0 | 1 | 5 |
| 16 | 2003 | 2020 | 31 | 49 | 17.50 | 1 | 3 | 3 | 1 | 8 |
| 17 | 2003 | 2020 | 51 | 68 | 17.25 | 2 | 3 | 3 | 1 | 8 |
| 18 | 2003 | 2020 | 35 | 52 | 17.33 | 0 | 3 | 3 | 4 | 7 |
| 19 | 2003 | 2019 | 31 | 48 | 16.25 | 1 | 7 | 1 | 3 | 11 |
| 20 | 2003 | 2019 | 20 | 37 | 16.42 | 1 | 4 | 3 | 1 | 9 |
| 21 | 2003 | 2020 | 36 | 53 | 17.08 | 0 | 3 | 3 | 2 | 6 |
| 22 | 2003 | 2019 | 27 | 43 | 16.33 | 4 | 0 | 2 | 0 | 6 |
| 23 | 2003 | 2019 | 33 | 50 | 16.92 | 2 | 3 | 4 | 0 | 7 |
| 24 | 2003 | 2020 | 32 | 49 | 17.42 | 1 | 1 | 4 | 0 | 6 |
| 25 | 2003 | 2020 | 29 | 46 | 17.33 | 1 | 4 | 6 | 0 | 10 |
| 26 | 2003 | 2019 | 24 | 40 | 16.50 | 2 | 6 | 5 | 5 | 17 |
| 27 | 2003 | 2020 | 30 | 47 | 17.33 | 3 | 1 | 4 | 2 | 9 |
| 28 | 2003 | 2019 | 38 | 54 | 16.42 | 4 | 2 | 3 | 1 | 9 |
| 29 | 2003 | 2020 | 35 | 52 | 17.17 | 2 | 4 | 3 | 1 | 8 |
| 30 | 2003 | 2019 | 30 | 47 | 16.17 | 4 | 3 | 6 | 1 | 11 |
| 31 | 2003 | 2020 | 29 | 47 | 17.58 | 4 | 1 | 6 | 2 | 11 |
| 32 | 2003 | 2020 | 27 | 44 | 16.92 | 1 | 6 | 3 | 2 | 12 |
| 33 | 2003 | 2020 | 28 | 45 | 17.17 | 2 | 6 | 5 | 3 | 13 |
| 34 | 2003 | 2019 | 32 | 48 | 16.17 | 2 | 3 | 1 | 1 | 6 |
| 35 | 2003 | 2020 | 34 | 52 | 17.67 | 1 | 5 | 2 | 3 | 8 |
| 36 | 2003 | 2019 | 25 | 42 | 16.58 | 2 | 2 | 2 | 3 | 8 |
| 37 | 2003 | 2019 | 29 | 45 | 16.50 | 2 | 2 | 3 | 0 | 6 |
| 38 | 2003 | 2017 | 50 | 65 | 14.33 | 0 | 1 | 3 | 0 | 4 |
| 39 | 2003 | 2010 | 45 | 53 | 7.42 | 0 | 1 | 0 | 0 | 1 |
| 40 | 2003 | 2014 | 51 | 62 | 11.25 | 1 | 3 | 1 | 0 | 5 |
| **Subject** | **Year start** | **Year end** | **Age start** | **Age end** | **Year duration** | **HCoV-NL63** | **HCoV-229E** | **HCoV-OC43** | **HCoV-HKU1** | **All HCoV** |
| 41 | 2003 | 2011 | 53 | 61 | 7.83 | 1 | 2 | 2 | 0 | 4 |
| 42 | 2005 | 2019 | 51 | 65 | 14.08 | 2 | 2 | 1 | 3 | 7 |
| 43 | 2003 | 2020 | 48 | 64 | 16.50 | 2 | 1 | 1 | 1 | 5 |
| 44 | 2003 | 2010 | 103 | 110 | 7.42 | 0 | 5 | 2 | 0 | 7 |
|  |  |  |  |  | **Total** | 79 | 132 | 143 | 52 | 364 |

**Supplementary Table 4** Association between HCoV infection and symptoms unadjusted and adjusted with age at follow up.

| **Symptom**^a^ | | **Any HCoV** | | | **HCoV-NL63** | | | **HCoV-229E** | | | **HCoV-OC43** | | | **HCoV-HKU1** | | |
| --- | --- | --- | --- | --- | --- | --- | --- | --- | --- | --- | --- | --- | --- | --- | --- | --- |
|  |  | *OR* | *(95% CI)* | *CM*^c^ | *OR* | *(95% CI)* | *CM*^c^ | *OR* | *(95% CI)* | *CM*^c^ | *OR* | *(95% CI)* | *CM*^c^ | *OR* | *(95% CI)* | *CM*^c^ |
| Fever | Unadjusted | 1.49 | (0.99-2.24) | 1% | *2.57* | *(1.37-4.82)* | *43%* | 1.13 | (0.59-2.19) | 16% | 1.29 | (0.71-2.37) | 11% | 2.00 | (0.88-4.53) | 19% |
|  | Adjusted^b^ | 1.47 | (0.89-2.43) |  | *1.80* | *(0.76-4.29)* |  | 1.34 | (0.65-2.81) |  | 1.45 | (0.72-2.93) |  | 2.47 | (0.95-6.43) |  |
| Cough | Unadjusted | **2.18** | **(1.39-3.41)** | **10%** | **3.05** | **(1.51-6.14)** | **13%** | **1.96** | **(1.02-3.77)** | **7%** | **2.15** | **(1.16-3.98)** | **16%** | 2.09 | (0.81-5.42) | 3% |
|  | Adjusted^b^ | **2.43** | **(1.41-4.21)** |  | **3.49** | **(1.50-8.13)** |  | **2.10** | **(0.98-4.52)** |  | **2.57** | **(1.26-5.25)** |  | 2.15 | (0.65-7.11) |  |
| Dyspnea | Unadjusted | 1.25 | (0.61-2.55) | NA | 1.57 | (0.47-5.18) | 32% | 1.32 | (0.48-3.68) | NA | 0.73 | (0.21-2.62) | NA | 0.90 | (0.14-5.74) | NA |
|  | Adjusted^b^ | NA | NA |  | 1.19 | (0.17-8.00) |  | NA | NA |  | NA | NA |  | NA | NA |  |
| Fatigue | Unadjusted | 1.39 | (0.98-1.98) | 3% | 1.57 | (0.84-2.94) | 18% | 1.07 | (0.61-1.89) | 9% | 1.62 | (0.99-2.63) | 23% | 1.03 | (0.43-2.49) | 20% |
|  | Adjusted^b^ | 1.43 | (0.96-2.15) |  | 1.92 | (0.96-3.85) |  | 1.17 | (0.63-2.17) |  | 1.32 | (0.75-2.36) |  | 1.28 | (0.49-3.31) |  |
| Headache | Unadjusted | 1.26 | (0.76-2.09) | 11% | 1.88 | (0.83-4.25) | 2% | 0.91 | (0.39-2.14) | 17% | 1.26 | (0.61-2.63) | 24% | 1.21 | (0.38-3.90) | 25% |
|  | Adjusted^b^ | 1.14 | (0.63-2.07) |  | 1.85 | (0.71-4.77) |  | 0.78 | (0.29-2.15) |  | 1.02 | (0.42-2.45) |  | 1.62 | (0.49-5.31) |  |
| Sore throat | Unadjusted | **1.50** | **(1.04-2.17)** | **11%** | 1.72 | (0.90-3.28) | 4% | 1.30 | (0.74-2.29) | 14% | 1.43 | (0.84-2.43) | 4% | 0.95 | (0.36-2.51) | 25% |
|  | Adjusted^b^ | **1.68** | **(1.11-2.52)** |  | 1.79 | (0.86-3.74) |  | 1.51 | (0.83-2.75) |  | 1.49 | (0.84-2.67) |  | 1.26 | (0.47-3.38) |  |
| Myalgia | Unadjusted | **2.54** | **(1.55-4.15)** | **7%** | 2.17 | (0.95-4.98) | 18% | **2.55** | **(1.32-4.94)** | **7%** | **2.70** | **(1.43-5.10)** | **3%** | **3.32** | **(1.38-8.01)** | **17%** |
|  | Adjusted^b^ | **2.73** | **(1.52-4.92)** |  | 2.65 | (1.03-6.83) |  | **2.73** | **(1.28-5.80)** |  | **2.63** | **(1.25-5.57)** |  | **4.02** | **(1.46-11.1)** |  |
| Nausea | Unadjusted | 1.12 | (0.65-1.94) | 22% | 1.77 | (0.75-4.20) | 75% | 1.28 | (0.59-2.79) | 7% | 0.37 | (0.10-1.35) | 16% | 1.41 | (0.45-4.40) | 27% |
|  | Adjusted^b^ | 0.92 | (0.50-1.69) |  | 1.01 | (0.32-3.15) |  | 1.20 | (0.53-2.71) |  | 0.44 | (0.13-1.44) |  | 1.11 | (0.29-4.30) |  |
| Diarrhea | Unadjusted | 1.16 | (0.73-1.85) | 36% | 0.99 | (0.39-2.53) | 46% | 0.94 | (0.44-1.99) | 22% | 1.31 | (0.68-2.52) | 21% | 1.34 | (0.49-3.69) | 58% |
|  | Adjusted^b^ | 0.85 | (0.48-1.48) |  | 0.68 | (0.21-2.22) |  | 0.77 | (0.33-1.79) |  | 1.08 | (0.52-2.25) |  | 0.85 | (0.22-3.33) |  |
| ^a^ Data points with no HCoV infection were assigned as the reference category. | | | | | | | | | | | | | | | | |
| ^b^ Age at each follow-up was stratified (< 35, 35 - 39, and ≥ 40) before inserted into the equation. The first age group was assigned as the reference category. | | | | | | | | | | | | | | | | |
| ^c^ CM (Confounding Magnitude) was calculated as follows: │OR_unadjusted_ - OR_adjusted_│/OR_adjusted_ | | | | | | | | | | | | | | | | |
| **Bold**: Association that remained significant after age adjustment. | | | | | | | | | | | | | | | | |
| *Italic*: Association that lose significance after age adjustment. | | | | | | | | | | | | | | | | |
| Underlined: Association that gained significance after age adjustment. | | | | | | | | | | | | | | | | |

**Supplementary Table 5** Percentage of reported symptom(s) at the end of reinfection interval of each and any endemic HCoV.

| **Symptom** | **Any HCoV** | | **HCoV-NL63** | | **HCoV-229E** | | **HCoV-OC43** | | **HCoV-HKU1** | |
| --- | --- | --- | --- | --- | --- | --- | --- | --- | --- | --- |
|  | *n* | *(%)* | *n* | *(%)* | *n* | *(%)* | *n* | *(%)* | *n* | *(%)* |
| N | 251 |  | 42 |  | 86 |  | 100 |  | 23 |  |
| Fever | 30 | (12%) | 8 | (19%) | 7 | (8%) | 10 | (10%) | 5 | (22%) |
| Cough | 31 | (12%) | 7 | (17%) | 9 | (10%) | 11 | (11%) | 4 | (17%) |
| Dyspnea | 5 | (2%) | 2 | (5%) | 1 | (1%) | 1 | (1%) | 1 | (4%) |
| Fatigue | 26 | (10%) | 4 | (10%) | 8 | (9%) | 14 | (14%) | 0 | (0%) |
| Headache | 11 | (4%) | 3 | (7%) | 2 | (2%) | 5 | (5%) | 1 | (4%) |
| Sore throat | 33 | (13%) | 6 | (14%) | 8 | (9%) | 17 | (17%) | 2 | (9%) |
| Myalgia | 20 | (8%) | 1 | (2%) | 7 | (8%) | 9 | (9%) | 3 | (13%) |
| Nausea | 10 | (4%) | 3 | (7%) | 4 | (5%) | 1 | (1%) | 2 | (9%) |
| Diarrhea | 15 | (6%) | 1 | (2%) | 5 | (6%) | 6 | (6%) | 3 | (13%) |
| Percentage was calculated based on absence/presence of any or each HCoV. | | | | | | | | | | |
| More than one symptom could be reported in one interval. | | | | | | | | | | |
